# Targeted ablation of signal transducer and activator of transduction 1 alleviates inflammation by microglia/macrophages and promotes long-term recovery after ischemic stroke

**DOI:** 10.1186/s12974-023-02860-4

**Published:** 2023-07-29

**Authors:** Wenxuan Han, Hongjian Pu, Sicheng Li, Yaan Liu, Yongfang Zhao, Mingyue Xu, Caixia Chen, Yun Wu, Tuo Yang, Qing Ye, Hong Wang, R. Anne Stetler, Jun Chen, Yejie Shi

**Affiliations:** 1 Pittsburgh Institute of Brain Disorders and Recovery and Department of Neurology, University of Pittsburgh, 3500 Terrace Street, S-510 BST, Pittsburgh, PA 15213 USA; 2 Department of Biostatistics, University of Pittsburgh, Pittsburgh, PA 15213 USA; 3 Geriatric Research, Education and Clinical Center, Veterans Affairs Pittsburgh Health Care System, Pittsburgh, PA 15261 USA

**Keywords:** Behavior test, Conditional gene knockout, Neuroinflammation, STAT1, White matter injury

## Abstract

**Background:**

Brain microglia and macrophages (Mi/MΦ) can shift to a harmful or advantageous phenotype following an ischemic stroke. Identification of key molecules that regulate the transformation of resting Mi/MΦ could aid in the development of innovative therapies for ischemic stroke. The transcription factor signal transducer and activator of transduction 1 (STAT1) has been found to contribute to acute neuronal death (in the first 24 h) following ischemic stroke, but its effects on Mi/MΦ and influence on long-term stroke outcomes have yet to be determined.

**Methods:**

We generated mice with tamoxifen-induced, Mi/MΦ-specific knockout (mKO) of STAT1 driven by Cx3cr1^CreER^. Expression of STAT1 was examined in the brain by flow cytometry and RNA sequencing after ischemic stroke induced by transient middle cerebral artery occlusion (MCAO). The impact of STAT1 mKO on neuronal cell death, Mi/MΦ phenotype, and brain inflammation profiles were examined 3–5 days after MCAO. Neurological deficits and the integrity of gray and white matter were assessed for 5 weeks after MCAO by various neurobehavioral tests and immunohistochemistry.

**Results:**

STAT1 was activated in Mi/MΦ at the subacute stage (3 days) after MCAO. Selective deletion of STAT1 in Mi/MΦ did not alter neuronal cell death or infarct size at 24 h after MCAO, but attenuated Mi/MΦ release of high mobility group box 1 and increased arginase 1-producing Mi/MΦ 3d after MCAO, suggesting boosted inflammation-resolving responses of Mi/MΦ. As a result, STAT1 mKO mice had mitigated brain inflammation at the subacute stage after MCAO and less white matter injury in the long term. Importantly, STAT1 mKO was sufficient to improve functional recovery for at least 5 weeks after MCAO in both male and female mice.

**Conclusions:**

Mi/MΦ-targeted STAT1 KO does not provide immediate neuroprotection but augments inflammation-resolving actions of Mi/MΦ, thereby facilitating long-term functional recovery after stroke. STAT1 is, therefore, a promising therapeutic target to harness beneficial Mi/MΦ responses and improve long-term outcomes after ischemic stroke.

**Supplementary Information:**

The online version contains supplementary material available at 10.1186/s12974-023-02860-4.

## Background

Ischemic stroke continues to be a significant health concern, even though it has dropped to the fifth leading cause of death in the United States. Currently, the only treatment option remains recanalization, but it only benefits a limited number of patients. Extensive efforts have been focused on developing neuroprotective agents over the past 30 years; however, all of the clinical trials stemming from these attempts ended in disappointment. This lack of success may be attributed to several factors, including a narrow focus on neurons without considering other cell types, the need to extend the treatment time window, and the absence of long-term functional outcomes in assessments [1]. Solely concentrating on protecting neurons from acute death may not be sufficient to prevent progressive injury and mitigate the subsequent deterioration of neurological functions after stroke. Therefore, there is a pressing need to develop treatments that provide longer-lasting effects to address these challenges effectively.

In the context of ischemic stroke, damaged neurons release danger-associated molecular patterns (DAMPs), which trigger inflammatory responses [2]. Inflammation is a process that is activated during the subacute stage of stroke and can persist for an extended period of time. This inflammatory response affects both the injury and repair processes in the post-stroke brain [2, 3]. Microglia and monocyte-derived macrophages, the innate immune cells in the brain, play critical roles in determining the inflammatory response and exert significant influence over the extent of injury and subsequent repair processes. Upon activation, microglia and macrophages (Mi/MΦ) can adopt detrimental or beneficial phenotypes that is proinflammatory or inflammation-resolving/pro-repair [4–6]. While this classification is well-described, only limited options are available to modulate the phenotype of Mi/MΦ to alter the inflammatory response and long-term functions in stroke. In this regard, one promising avenue for intervention is targeting the signal transducer and activator of transcription (STAT) family of transcription factors.

STATs can be phosphorylated by the Janus kinases following ligand–receptor interactions, translocate to the nucleus, and bind to specific DNA sequence motifs [7]. As transcription regulators, STATs have been implicated in a wide range of cellular processes, including apoptosis and inflammation [8]. Specifically, STAT1 is potently activated in canonical interferon signaling and directly induces proinflammatory factors, such as inducible nitric oxide synthase (iNOS) and interleukin (IL)-12 [7]. Despite its critical role in immune and inflammatory responses, studies on STAT1 in ischemic stroke have primarily focused on its pro-apoptotic action on neurons at the acute injury stage (the first 24 h), whereby STAT1 knockout mice were reported to develop smaller brain infarct and have less neuronal apoptosis at 24 h after brain ischemia [9]. The use of the global STAT1 knockout mice in these studies, however, does not allow for the distinction between the contributions of neurons and non-neuronal cells to the observed effects. Furthermore, there have been no studies conducted to assess the impact of STAT1 knockout on long-term stroke outcomes. It remains unknown whether STAT1 regulates Mi/MΦ function and affects the evolving long-term neurological recovery—the primary endpoint of clinical stroke. On this backdrop, the present study aimed to directly investigate the specific role of STAT1 in the modulation of Mi/MΦ phenotype, inflammation, and long-term functional recovery after ischemic stroke, using a Mi/MΦ-targeted STAT1 knockout mouse model.

## Methods

Key resources that are essential to reproduce the results are provided in Additional file 1: Table S1.

### Animals

C57BL/6J (RRID: IMSR_JAX:000664), STAT1^LoxP^ (RRID: MMRRC_032054-JAX) [10], and Cx3cr1^CreER^ (RRID: IMSR_JAX:021160) [11] mice were purchased from the Jackson Laboratory (Additional file 1: Table S1). The STAT1^LoxP^ mice harbor a conditional null allele of the *Stat1* gene, where *LoxP* sites flank its first two untranslated exons and the first translated exon. Mi/MΦ-specific STAT1 knockout (STAT1 mKO) mice were obtained by crossing the Cx3cr1^CreER^ mice and STAT1^LoxP^ mice for two generations. The STAT1 mKO mice (genotype: *Cx3cr1*^*CreER/wt*^*; Stat1*^*flox/flox*^) were viable, fertile, and did not exhibit any gross physical or behavioral abnormalities. To induce gene deletion, STAT1 mKO mice received intraperitoneal injections of tamoxifen (75 mg/kg daily for 4 days). Hemizygous Cx3cr1^CreER^ mice (genotype: *Cx3cr1*^*CreER/wt*^*; Stat1*^*wt/wt*^) served as age- and sex-matched wild type (WT) control mice for the STAT1 mKO mice and received the same tamoxifen treatments. Mice were subjected to experimental stroke 10 days after tamoxifen treatments.

Mice were housed in a specific pathogen-free facility with a 12-h light/dark cycle. Food and water were available ad libitum. All animal experiments were performed in accordance with the NIH *Guide for the Care and Use of Laboratory Animals*, approved by the University of Pittsburgh Institutional Animal Care and Use Committee, and reported in accordance with the ARRIVE guidelines [12]. Experimental procedures were performed following criteria derived from Stroke Therapy Academic Industry Roundtable (STAIR) group guidelines for preclinical evaluation of stroke therapeutics [13]. Accordingly, experimental group assignments were randomized with a lottery-drawing box, and surgeries and all outcome assessments were performed by investigators blinded to mouse genotype and experimental group assignment whenever possible.

### Transient focal cerebral ischemia model

Transient focal cerebral ischemia was induced in adult male and female mice (10–14 weeks, 22–28 g) by intraluminal occlusion of the left middle cerebral artery (MCA) for 1 h, as we previously described [14]. Three weeks before MCA occlusion (MCAO), female mice were subjected to bilateral ovariectomy as we described previously [15], which depletes endogenous estrogen and partially reproduces the systemic conditions in postmenopausal women. Mice were anesthetized with 1.5% isoflurane in 67%:30% N_2_O/O_2_. A 7–0 nylon monofilament with silicone–rubber coated tip (Doccol Corporation 702,134; tip diameter 0.21 mm, coating length 3–4 mm) was introduced into the internal carotid artery, advanced to the origin of the MCA, and left in place for 1 h. Rectal temperature was maintained at 37 ± 0.5 °C with a heating pad during surgical procedures. Regional cerebral blood flow (rCBF) was monitored in all stroke animals using laser Doppler flowmetry or a two-dimensional laser speckle imaging system (PeriCam PSI-NR; Perimed) as we previously described [14]. Only animals with an rCBF reduction of > 70% of pre-MCAO baseline levels were included for further experimentation. Animals that died during or immediately after surgery were excluded from the studies (less than 2%). Sham-operated animals underwent the same anesthesia and surgical procedures with the exception of the MCA occlusion.

### Neurobehavioral tests

The rotarod test, cylinder test, foot fault test, and Morris water maze test were performed to assess neurological functions before and up to 35 days after MCAO. The mice that died before 35 days after MCAO were excluded from the final behavioral analyses. On the first day after MCAO, neurological functions were evaluated by a six-point scale [16] as follows: 0, no observable deficit; 1, torso flexion to right; 2, spontaneous circling to right; 3, leaning/falling to right; 4, no spontaneous movement; 5, death.

#### Rotarod test

The rotarod test was performed to assess post-stroke motor deficits as we described previously [17]. Briefly, mice were placed on a rotating drum with a speed accelerating from 4 to 40 rpm within 5 min. The time at which the mouse fell off the drum (latency to fall) was recorded. On the day of MCAO surgery (but before the surgery), 5 trials were performed on each mouse and the mean of trial numbers 3, 4, and 5 was used as the pre-MCAO baseline value. After MCAO, mice were tested for 5 trials on each testing day with intervals of 15 min, and the data for trial numbers 3–5 were used to calculate the mean latency to fall on that day.

#### Cylinder test

The cylinder test was performed to assess forepaw use asymmetry after stroke as we described previously [16]. The mouse was placed in a transparent cylinder (diameter: 9 cm; height: 15 cm), and videotaped from the top for 8 min. Videotapes were analyzed in slow motion, and forepaw (left/right/both) use during the first contact against the cylinder wall after rearing and during lateral exploration was recorded. Uninjured mice typically do not exhibit any inclination toward one forepaw over the other, whereas mice that have suffered a stroke exhibit reduced utilization of their right forepaw (impaired side; contralateral to ischemic lesion), depending on the extent of the injury. Forepaw use asymmetry was calculated with the following equation: contralateral paw use = right/(left + right + both) × 100%.

#### Foot fault test

The foot fault test was performed as we described previously [18] to assess sensorimotor coordination. Mice were placed on an elevated grid surface with a grid opening of 2.25 cm^2^ and videotaped for 2 min from below the grid. The videotapes were analyzed by a blinded investigator to count the number of total steps and the number of foot faults made by the right limbs (impaired side; contralateral to ischemic lesion). Foot faults were recorded when the mouse misplaced its left forepaw or hindpaw, such that the paw fell through the grid, and expressed as a percentage of total steps.

#### Morris water maze test

The Morris water maze test was performed as we described previously [14] to assess cognitive functions. Briefly, a round platform (diameter: 11 cm) was submerged 1.5 cm under the water surface in the center of one quadrant of a circular pool (diameter: 109 cm; depth: 33 ± 0.5 cm). White tempera paint was added to the water for opacity and the temperature of the pool was maintained at 20 ± 1 °C. The test was composed of a spatial acquisition phase to assess learning capacity and a spatial retention phase to assess memory function. Spatial learning was assessed 22–26 days after MCAO, where the mouse was released from one of the four quadrants and was allowed to swim for 60 s to find the hidden platform. Mice were pre-trained for 3 consecutive days before MCAO (4 trials on each day). After MCAO, four trails were performed on each testing day. At the end of each trial, the mouse was placed on the platform or was allowed to remain on the platform for 30 s with prominent spatial cues displayed around the room. The time spent finding the hidden platform was recorded as the latency to escape from the forced swimming task, and the mean latency of four trials was quantified as a measure of spatial learning. Spatial memory was evaluated on day 27 after MCAO by removing the hidden platform. Each mouse was placed in the pool for a single 60-s probe trial. The time the mouse spent in the target quadrant were recorded as spatial memory and expressed as percentage of the total trial time (60 s).

### Flow cytometry and fluorescence-activated single cell sorting (FACS)

Mice were deeply anesthetized and transcardially perfused with ice-cold Hank’s balanced salt solution, and the ipsilesional and non-injured contralesional cerebral hemispheres were harvested. Single-cell suspensions were prepared from the mouse brain using the *Neural Tissue Dissociation Kit* and gentleMACS Octo Dissociator with Heaters (Miltenyi Biotec) according to the manufacturer’s instructions and as we described previously [19]. Suspensions were passed through a 70 μm cell strainer, and fractionated on a 30% and 70% Percoll gradient at 800 g for 30 min to remove myelin and cell debris. Mononuclear cells at the interface were collected and resuspended at 1 × 10^7^ cells per mL. The cells were first stained with the *Zombie NIR Fixable Viability Dye* for 20 min at 4 °C for assessment of viability. The cells were then stained with fluorophore-conjugated antibodies (Additional file 1: Table S1) recognizing surface antigens. After washing, cells were fixed and permeabilized with an eBioscience *Foxp3/Transcription Factor Staining Buffer Set* or 4% formaldehyde followed by ice-cold methanol, and then incubated with antibodies recognizing intracellular antigens. Flow cytometry was performed using a BD LSRFortessa Cell Analyzer driven by the FACSDiva software or a Cytek Aurora spectral analyzer driven by the SpectroFlo software. FACS was performed using a BD FACSAria cell sorter driven by the FACSDiva software. Fluorescence compensation was performed using single-stained *OneComp eBeads* according to the manufacturer’s instructions.

Data were analyzed using the FlowJo software to quantify positively stained cells. Furthermore, individual immune cells (CD45^+^ gated) were plotted using *tSNE* function in FlowJo to map high-dimensional cytometry data onto two dimensions based on the *fast interpolation-based t-distributed stochastic neighbor embedding (FIt-SNE)* algorithm [20]. t-SNE plots were generated with 1000 iterations of 6000 cells randomly selected from each mouse. Major cell types were identified according to their expression of prototypic markers.

### Real-time polymerase chain reaction (PCR)

Quantitative PCR was performed on FACS-sorted cells to verify the deletion of STAT1. Total RNA was extracted from the sorted cells using a Qiagen *RNeasy Plus Micro Kit*, and genomic DNA was eliminated. First-stand cDNA was generated using a Qiagen *RT2 First Strand Kit* according to the manufacturer's instructions. Real-time PCR was performed using the Qiagen *RT2 SYBR Green qPCR Mastermix* on a Bio-Rad Opticon 2 Real-Time PCR Detection System. All reactions were performed in triplicate, and the amount of mRNA was normalized to internal controls (*Gapdh*). Data were summarized as fold changes relative to WT control mice.

### Immunohistochemistry and data analyses

Mice were deeply anesthetized and transcardially perfused with 0.9% NaCl, followed by 4% paraformaldehyde in PBS. Brains were harvested and cryoprotected in 30% sucrose in PBS, and frozen serial coronal brain sections (25-μm thick) were prepared using a ThermoFisher HM525 NX cryostat. Sections were blocked with 5% donkey serum in PBS for 1 h, followed by overnight incubation (at 4 °C) with primary antibodies (Additional file 1: Table S1). For immunostaining involving mouse primary antibodies, a *Mouse on Mouse Immunodetection Kit* was used following the manufacturer’s instructions. After washing, sections were incubated for 1 h at 20 °C with donkey secondary antibodies conjugated with DyLight 488 or Cy3 fluorophores (1:1000, Jackson ImmunoResearch Laboratories). Alternate sections from each experimental condition were incubated in all solutions except the primary antibodies to assess nonspecific secondary antibody staining. To visualize apoptotic cells, TUNEL staining was performed using a Roche In Situ Cell Death Detection Kit following manufacturer’s instructions. Sections were then mounted and coverslipped with Fluoromount-G containing DAPI (Southern Biotech). Fluorescence images were captured with an inverted Nikon Diaphot-300 fluorescence microscope equipped with a SPOT RT slider camera and Meta Series Software 5.0 (Molecular Devices), or with an Olympus Fluoview FV1000 confocal microscope and FV10-ASW 2.0 software.

Quantitative analysis of immunofluorescence staining images was performed manually by a blinded investigator using ImageJ. Brain infarct and tissue loss after MCAO were measured on six equally spaced coronal brain sections encompassing the MCA territory immunostained for microtubule-associated protein 2 (MAP2). Volumes of infarct or tissue loss were calculated as the volume of the MAP2-immunopositive contralateral hemisphere minus that of the ipsilateral hemisphere. Positively stained cells were manually counted from 1 to 2 randomly selected 40 × microscopic fields (317.4 μm × 317.4 μm) in the cortex and striatum for each brain section, and 2 brain sections were processed for each mouse. Nodes of Ranvier (NORs) were counted from two 60 × microscopic fields with 2.5 × digital magnification (final area 83.97 μm × 83.97 μm) in the external capsule. A threshold was set to differentiate the target signal from background.

The image-processing software Imaris was used to reconstruct three-dimensional images of Iba1, CD16/32, and arginase 1 (Arg1) immunofluorescence and quantify morphological parameters of cells as we previously described [15]. Briefly, a z-stack of 5 images spanning 10 μm were imported into Imaris, and the immunosignal of each channel was remodeled to 3D images by the *surface* operation. Smoothing was set at 0.6 μm for all channels and images. A threshold was set to differentiate the target signal from background. Non-specific signals were manually removed, and the 3D-rendered images were constructed. All images were processed with the same adjustments. Morphological analysis of resting microglia was performed on 3 randomly selected regions of interest per brain. Cell volume and sphericity were automatically calculated by Imaris.

### RNA sequencing (RNA-seq)

Microglia were gated as CD11c^–^Ly6G^–^CD11b^+^CD45^low^ cells and enriched from the mouse brain by FACS as described above, and subjected to bulk RNA-seq. RNA extraction, library preparation, and sequencing were performed at the University of Pittsburgh HSCRF Genomics Research Core as we previously described [18]. Briefly, total RNA was extracted from FACS-sorted cells using a Qiagen RNeasy Plus Micro Kit, and RNA integrity was assessed using the High Sensitivity RNA ScreenTape system on an Agilent 2200 TapeStation. The SMART-Seq HT Kit was used to generate cDNA from 10 ng of total RNA, and the cDNA product was checked by an Agilent Fragment Analyzer system for quality control. The sequencing library was constructed by following the Illumina Nextera XT Sample Preparation Guide. One nanogram of input cDNA was tagmented (tagged and fragmented) and amplified using the Illumina Nextrera XT kit. Sequence libraries of each sample were finally equimolarly pooled and sequenced on an Illumina Nextseq 500 system, using a paired-end 75-bp strategy.

### RNA-seq data analysis

RNA-seq data were analyzed using R/Bioconductor [21], following our published pipeline [18, 22]. Preprocessing of the RNA-seq data was completed using Chipster [23]. Fastq files were quality controlled using FastQC [24], and all samples passed quality control criteria. Reads were mapped to the GRCm38 mouse genome using HISAT2 [25] and counted by HTSeq [26]. Genes were identified by Ensembl ID [27]. The R package DESeq2 [28] was used to normalize the counts and to perform differential expression analysis. Differentially expressed genes (DEGs) were defined as genes with a log_2_(fold change) > 1 or < − 1, and with a false discovery rate (FDR) adjusted *p* value < 0.05 (Benjamini–Hochberg method).

DEGs identified by DESeq2 were submitted to Ingenuity Pathway Analysis (IPA) for pathway analysis using the Ingenuity Knowledge Base (Qiagen Bioinformatics). The fold change and adjusted *p* value for each gene were used to perform the core analysis. Diseases and functions were considered significantly enriched with a *p* value of overlap < 0.01 and an activation *z*-score > 2 (predicted to be activated) or < − 2 (predicted to be inhibited). The *Upstream Regulator* analysis was used to identify the cascade of upstream transcriptional regulators that can explain the observed gene expression changes in the data set. An upstream regulator was predicted to be strongly activated or inhibited if its activation z-score was > 2 or < − 2, respectively. The cutoff *p* value was set at 0.01.

### Proteomic array analysis

Mice were deeply anesthetized and transcardially perfused with 0.9% NaCl, and fresh brain tissues were harvested. Protein was extracted from the left cerebral hemisphere using cell lysis buffer with protease and phosphatase inhibitor cocktail. The concentration of protein was measured using the Bradford protein assay. The content of 40 inflammatory factors was measured using a RayBiotech *Mouse Inflammation Array* kit following manufacturer’s instructions and as we described previously [19]. The signal intensity for each antigen-specific antibody spot was measured using ImageJ, and background signal averaged from 3 blank spots was subtracted. The signal intensity for each inflammatory factor was then normalized to the average intensity of the 3 positive control spots on each membrane. The concentrations of various factors were expressed as fold changes relative to sham controls.

### Statistical analyses

Data are presented as mean ± standard deviation (SD). Individual data points are plotted where applicable. Statistical comparison between two groups was accomplished by the student’s *t* test (for normally distributed data) or Mann–Whitney *U* test (for non-normally distributed data). Differences in means among multiple groups were analyzed by one- or two-way ANOVA followed by the Bonferroni-adjusted multiple comparisons. Data that do not follow a normal distribution were analyzed by Kruskal–Wallis test followed by the Dunn's test. Data from behavior tests which consist of repeated testing on different days were analyzed by two-way repeated measures ANOVA. Linear regression analysis and Pearson’s correlation coefficients were used to correlate the histological parameters with neurobehavioral functions. A p value less than 0.05 was deemed statistically significant, and all testing was two-tailed.

## Results

### STAT1 is activated in brain Mi/MΦ after ischemic stroke

**Fig. 1:**
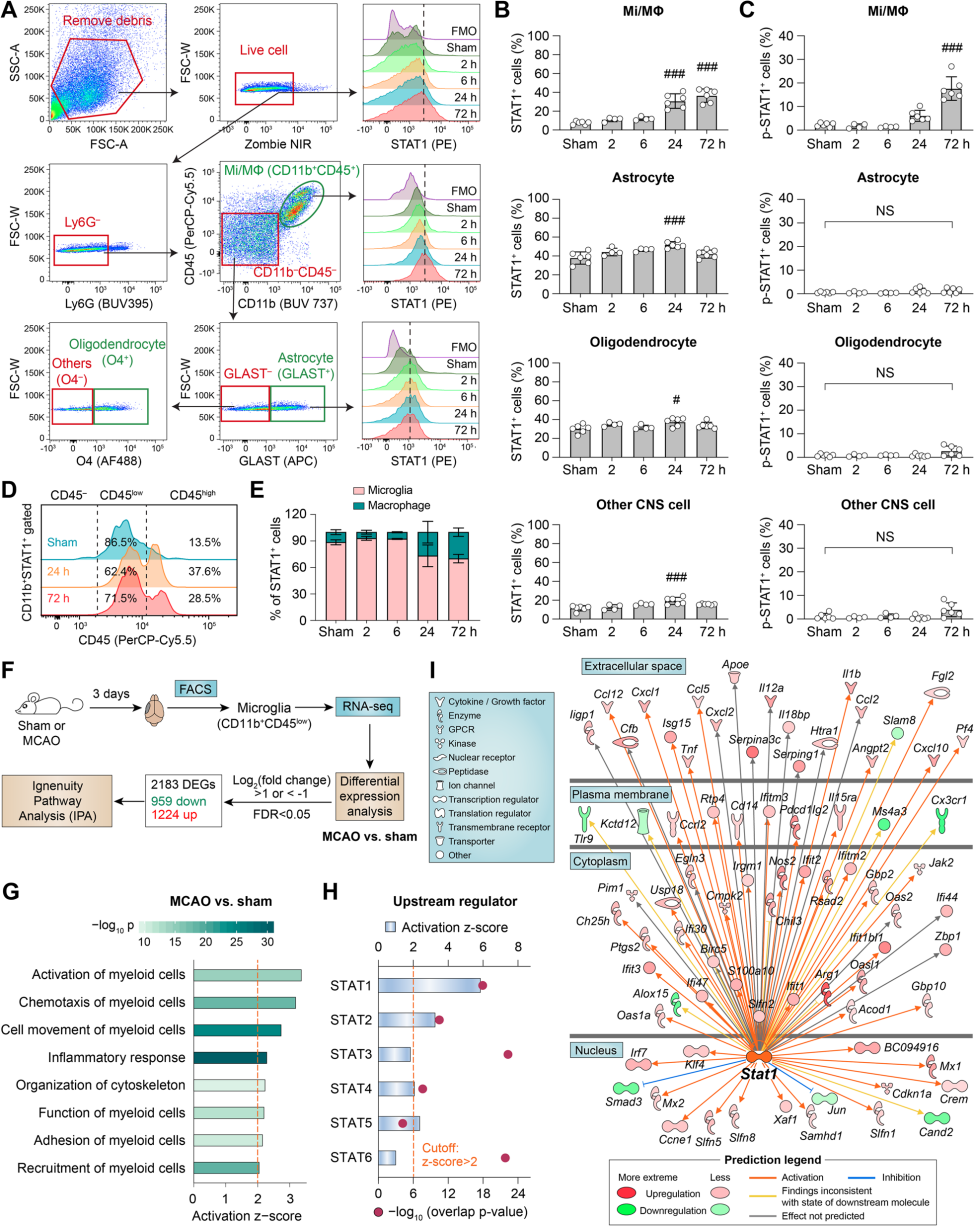
STAT1 is activated in brain microglia/macrophages after ischemic stroke. **A**–**E** Mice were subjected to 1-h MCAO or sham injury. Expression of STAT1 in various types of cells in the brain was examined at 2, 6, 24, and 72 h after MCAO using flow cytometry. **A** Gating strategy for microglia/macrophages (Mi/MΦ; Ly6G^–^CD11b^+^CD45^+^), astrocytes (GLAST^+^), oligodendrocytes (O4^+^), and other CNS cells (CD11b^–^CD45^–^GLAST^–^O4^–^). STAT1-immunopositive cells were gated based on the Fluorescence Minus One (FMO) control. **B**, **C** The numbers of cells immunopositive for STAT1 **B** or phosphorylated STAT1 **C** were quantified in Mi/MΦ, astrocytes, oligodendrocytes, and other CNS cells, and were expressed as percentages of all cells of that type. **D** Cells co-expressing CD11b and STAT1 were examined for their expression of CD45. CD45^+^ cells were classified as microglia (CD45^low^) and macrophages (CD45^high^). **E** Percentages of microglia and macrophages in all CD11b^+^STAT1^+^ cells at different times after MCAO. More than 60% of STAT1^+^ cells were microglia in all groups. *n* = 4–6 mice/group. ^#^*p* < 0.05, ^###^*p* < 0.001 vs. sham. NS, no significant difference. **F** RNA-seq experimental design and data analysis pipeline. Mice were subjected to MCAO or sham injury. Three days later, microglia were purified from the brain by fluorescence-activated cell sorting (FACS) and subjected to RNA-seq. *n* = 2–3 biological replicates/group. Ingenuity pathway analysis (IPA) was performed on the differentially expressed genes (DEGs) induced by MCAO compared to sham controls. **G** IPA predicted several functions of myeloid cells to be significantly activated (*z*-score > 2, *p* value of overlap < 0.01) in post-MCAO microglia. **H** The upstream regulator analyses in IPA calculated the activation z-score and *p* value of overlap for each STAT as a potential upstream regulator in post-MCAO microglia. The cutoff values for predicated activation were *z*-score > 2 and *p* value < 0.01. **I** DEGs that are regulated by STAT1 are shown in a network view, with annotations on the subcellular localization and types of their products

It has been reported that ischemic stroke can induce the activation of STAT1 in the brain [9], yet the cell type(s) accountable for the increased activity of STAT1 have not been investigated thus far. We performed flow cytometry to assess the expression of STAT1 in various types of cells in the mouse brain after transient focal cerebral ischemia induced by 1 h of MCAO (Fig. 1A). Robust upregulation of STAT1 was observed in CD11b^+^ Mi/MΦ at 24 and 72 h after MCAO compared to control mice subjected to sham injury, whereby the percentages of Mi/MΦ expressing STAT1 increased by 3 and 3.8 folds, respectively (Fig. 1B). In contrast, a larger portion of astrocytes, oligodendrocytes and other CNS cells expressed STAT1 under baseline conditions compared to Mi/MΦ, but these cell types did not exhibit substantial alterations in response to ischemic stroke (Fig. 1B). On the other hand, phosphorylation of STAT1 at Y701 (reflecting its activity) markedly increased in Mi/MΦ at 24 and 74 h after MCAO, whereas astrocytes, oligodendrocytes and other CNS cells had low levels of phospho-STAT1 from 2 to 72 h after MCAO (Fig. 1C). We conducted further classification of all CD11b^+^ cells expressing STAT1 based on their expression of CD45 (Fig. 1D), and found that more than 60% of STAT1-expressing cells were CD45^low^ (microglia) at all timepoints examined (Fig. 1E). The proportion of CD45^high^ cells (macrophages) in STAT1-immunopositive cells gradually increased from 2 to 72 h after MCAO (Fig. 1E), likely reflecting the infiltration of blood monocytes/macrophages into the post-stroke brain. Together, these data suggest that STAT1 is elevated and activated in post-stroke Mi/MΦ at the subacute stage (24 and 72 h) and may influence the function of these cells.

To confirm the activation of STAT1 in Mi/MΦ after stroke, we conducted RNA-seq analyses on microglia enriched from the post-stroke brain 3 days after MCAO and from non-injury sham controls using FACS (Fig. 1F). Differential expression analysis identified 2183 genes that were upregulated or downregulated with a Log_2_(fold change) > 1 or < − 1 and an FDR-adjusted *p* value < 0.05 in post-MCAO microglia compared to sham microglia (Additional file 2: Table S2), suggesting that microglia undergo substantial transcriptome reprogramming in response to ischemic brain injury. To gain further insight into the biological functions implicated by these DEGs, we performed IPA and examined the diseases and functions overrepresented by the DEGs. Functions related to myeloid cells and inflammatory responses were predicted to be strongly activated (*z*-score > 2 and *p* < 0.01) in post-MCAO microglia (Fig. 1G). Furthermore, *Upstream Regulator* analysis predicted several members of the STAT family to be activated in post-stroke microglia, with STAT1 being the most activated (Fig. 1H; *z*-score = 5.85, *p* = 9.84 × 10^–19^). A total of 81 DEGs were in the molecular network of STAT1 targets (Fig. 1I), including genes encoding the proinflammatory cytokines IL-1β, tumor necrosis factor alpha (TNF-α), and IL-12, all of which were upregulated in microglia after ischemic stroke. The collective findings from our flow cytometry and RNA-seq analyses provide strong evidence that STAT1 is activated in brain Mi/MΦ after ischemic stroke, and may dictate the proinflammatory responses of Mi/MΦ at the subacute injury stage.

### In vivo* s*elective deletion of STAT1 does not induce changes to homeostatic microglia

**Fig. 2:**
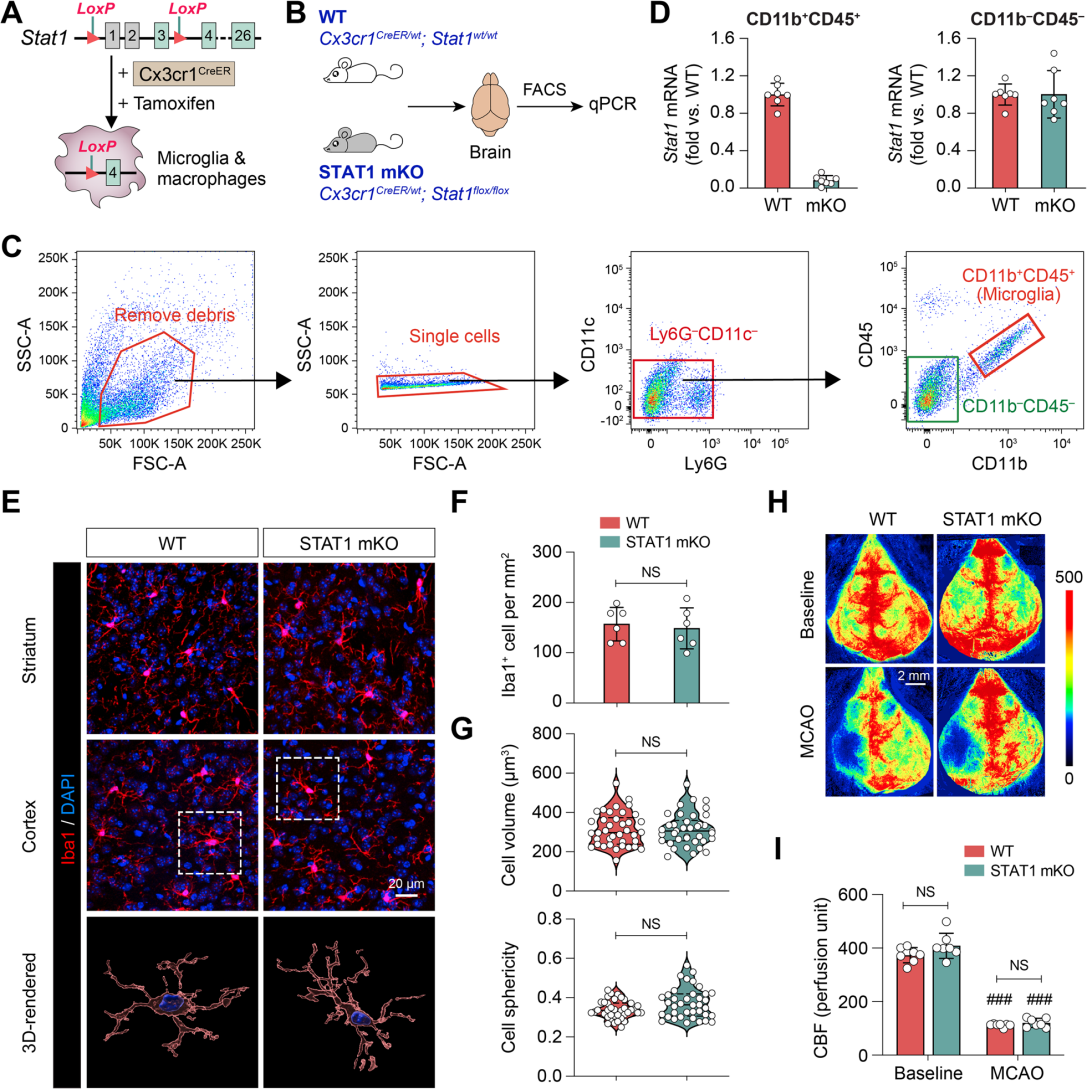
Generation and characterization of microglia/macrophage-targeted STAT1 knockout (STAT1 mKO) mice. **A** Generation of STAT1 mKO mice. *LoxP* sites were engineered flanking the first two untranslated exons and the first translated exon of the *Stat1* gene. When crossed to Cx3cr1^CreER^ mice, the *LoxP* sites recombine and delete the floxed region of *Stat1*, knocking out the protein in CX3CR1-expressing cells in the presence of tamoxifen. **B**–**D** Microglia (CD11b^+^CD45^+^ cells) and other CNS cells (CD11b^–^CD45^–^ cells) were purified from the brains of STAT1 mKO mice and WT mice by FACS, and subjected to quantitative PCR to measure the mRNA levels of *Stat1*. *n* = 7 mice/group. **E** The morphology of microglia was assessed by Iba1 immunostaining in the striatum and cortex of STAT1 mKO mice and WT mice. Cells were counterstained with DAPI for nuclear labeling. *Rectangle*: the region enlarged and 3D-rendered in the 3rd row. **F**, **G** STAT1 mKO did not alter the number **F** or morphology **G** of microglia in the homeostatic brain. *n* = 6 mice/group **F** or 38 cells/group (**G**). **H**, **I** Focal cerebral ischemia was induced in STAT1 mKO mice and WT control mice by 1 h of MCAO. **H** Two-dimensional laser speckle images show cortical CBF before MCAO (baseline) and 10 min after the onset of MCAO. **I** Summarized data show that STAT1 mKO mice had comparable cortical CBF changes during MCAO with WT mice. *n* = 7 mice/group. ^###^*p* < 0.001 MCAO vs. baseline. *NS* no significant difference

To date, there have been no studies that directly address the role of STAT1 in Mi/MΦ due to the lack of specific genetic models targeting these cell types. To bridge this knowledge gap, we generated mice with tamoxifen-induced, Mi/MΦ-targeted knockout of STAT1 (STAT1 mKO) by crossing the Cx3cr1^CreER^ mice with STAT1^LoxP^ mice (Fig. 2A). To confirm microglia-targeted deletion of STAT1, we isolated microglia (CD11b^+^CD45^+^ cells) and other CNS cells (CD11b^–^CD45^–^ cells) from the brains of STAT1 mKO mice and age-matched WT control mice 10 days after tamoxifen treatment and performed quantitative PCR (Fig. 2B, C). mRNA of the *Stat1* gene was reduced by 92% in microglia from STAT1 mKO mice compared to WT controls, whereas its levels were similar in non-microglial CNS cells between STAT1 mKO and WT mice (Fig. 2D), confirming selective deletion of STAT1 in microglia.

The STAT1 mKO mice did not display any noticeable physical or behavioral abnormalities when compared to WT control mice. To assess whether STAT1deletion in microglia had any discernible effects on microglia abundance or morphology under physiological conditions, we performed immunostaining of Iba1 on brain sections from naïve STAT1 mKO mice and WT mice (Fig. 2E). STAT1 mKO did not cause changes in the number of Iba1^+^ cells (Fig. 2F) or the volume and sphericity of these cells (Fig. 2G), suggesting that loss of STAT1 did not result in overt phenotypic changes of homeostatic microglia. Furthermore, when subjected to transient focal cerebral ischemia induced by MCAO, STAT1 mKO mice and WT mice had comparable rCBF reduction (Fig. 2H, I), suggesting that they had similar hemodynamics in response to ischemic injury.

### Mi/MΦ-targeted STAT1 deletion does not reduce acute neuronal cell death after ischemic stroke

**Fig. 3:**
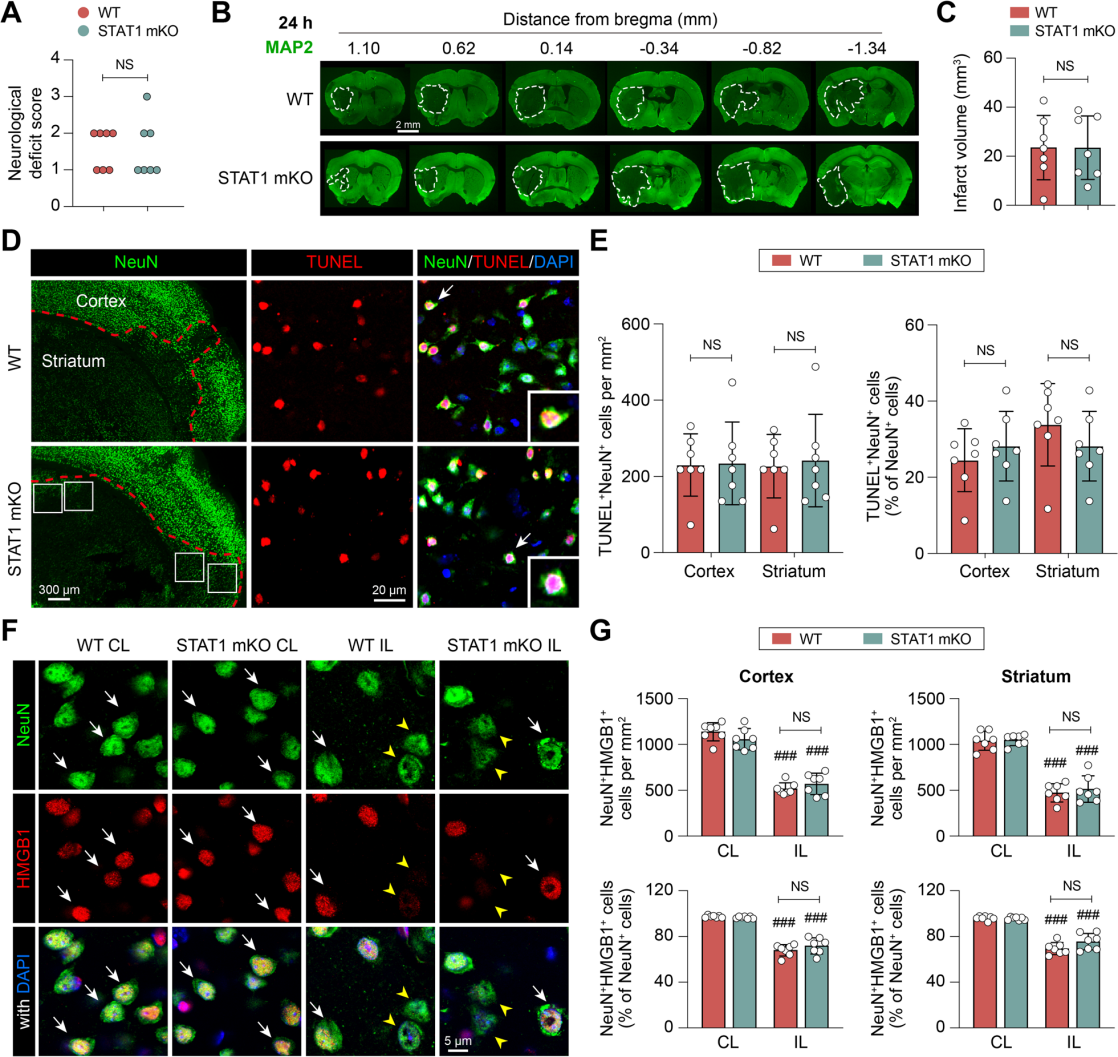
Selective STAT1 deletion in microglia/macrophages does not reduce acute neuronal death after ischemic stroke. STAT1 mKO mice and WT control mice were subjected to 1-h MCAO followed by 24 h of reperfusion. **A** Neurological deficit scores. **B**, **C** Brain infarct volume was measured on six coronal sections encompassing the MCA territory immunostained for MAP2 (*green*). **D** TUNEL/NeuN double-staining was performed to assess the apoptosis of neurons. *Dashed line*: infarct border. *Arrows*: apoptotic neurons (TUNEL^+^NeuN^+^ cells) enlarged in the insets. **E** The numbers of apoptotic neurons in the inner border zone (*rectangles* in **D**) in the cortex and striatum. **F** Neuronal expression of HMGB1 was examined in the ipsilateral (IL) inner border zone or corresponding areas in the non-injured contralateral (CL) side by double-label immunostaining of NeuN and HMGB1. *Arrow*: NeuN and HMGB1 double-immunopositive cells. *Arrowhead*: NeuN^+^ cells that are negative for HMGB1. **G** The numbers of NeuN^+^HMGB1^+^ cells were counted in the cortex and striatum, and expressed as numbers per mm^2^ (*upper panels*) or as percentages of total NeuN^+^ cells (*lower panels*). *n* = 7 mice/group. ^###^*p* < 0.001 IL vs. CL. *NS* no significant difference

Previous studies have found that constitutive STAT1 knockout reduces brain infarct volume 24 h after ischemic stroke, mainly through protection of neurons against acute apoptosis [9]. We, therefore, examined whether selective deletion of STAT1 only in Mi/MΦ had an impact on acute brain injury after ischemic stroke. Male STAT1 mKO mice and WT control mice were subjected to 1-h MCAO followed by 24 h of reperfusion. At 24 h after MCAO, STAT1 mKO mice had similar neurological deficit scores with WT controls (Fig. 3A), and their infarct volumes were not different as measured by immunostaining of the neuronal marker MAP2 (Fig. 3B, C). On a microscopic level, the numbers of apoptotic neurons identified by double-staining of TUNEL and NeuN (Fig. 3D) were also similar between STAT1 mKO mice and WT mice in both the peri-infarct cortex and striatum (Fig. 3E).

Dead/dying neurons in the post-stroke brain release DAMPs to elicit proinflammatory responses [2, 29]. We examined the expression of high mobility group box 1 (HMGB1), a major DAMP in ischemic brain [30, 31], in neurons at 24 h after MCAO. In the non-injured contralateral brain hemisphere, most NeuN^+^ cells (> 95%) were also immunopositive for HMGB1 (Fig. 3F), indicating healthy neurons retaining endogenous HMGB1. On the contrary, 32.0% and 30.5% of NeuN^+^ cells became HMGB1-negative in the ipsilateral cortex and striatum of WT mice, respectively (Fig. 3G), suggesting that these neurons were damaged and released HMGB1 into the extracellular space. The numbers of HMGB1^+^ neurons were similar in STAT1 mKO mice and WT mice 24 h after MCAO (Fig. 3 F and G), suggesting similar neuronal injury and the initial trigger of proinflammatory responses at the acute stage after ischemic stroke. Collectively, these data suggest that targeted deletion of STAT1 only in Mi/MΦ does not affect neuronal death or stroke outcome at the acute injury stage (24 h after MCAO).

### STAT1 deficiency ameliorates neurotoxic Mi/MΦ responses at the subacute stage after ischemic stroke

**Fig. 4:**
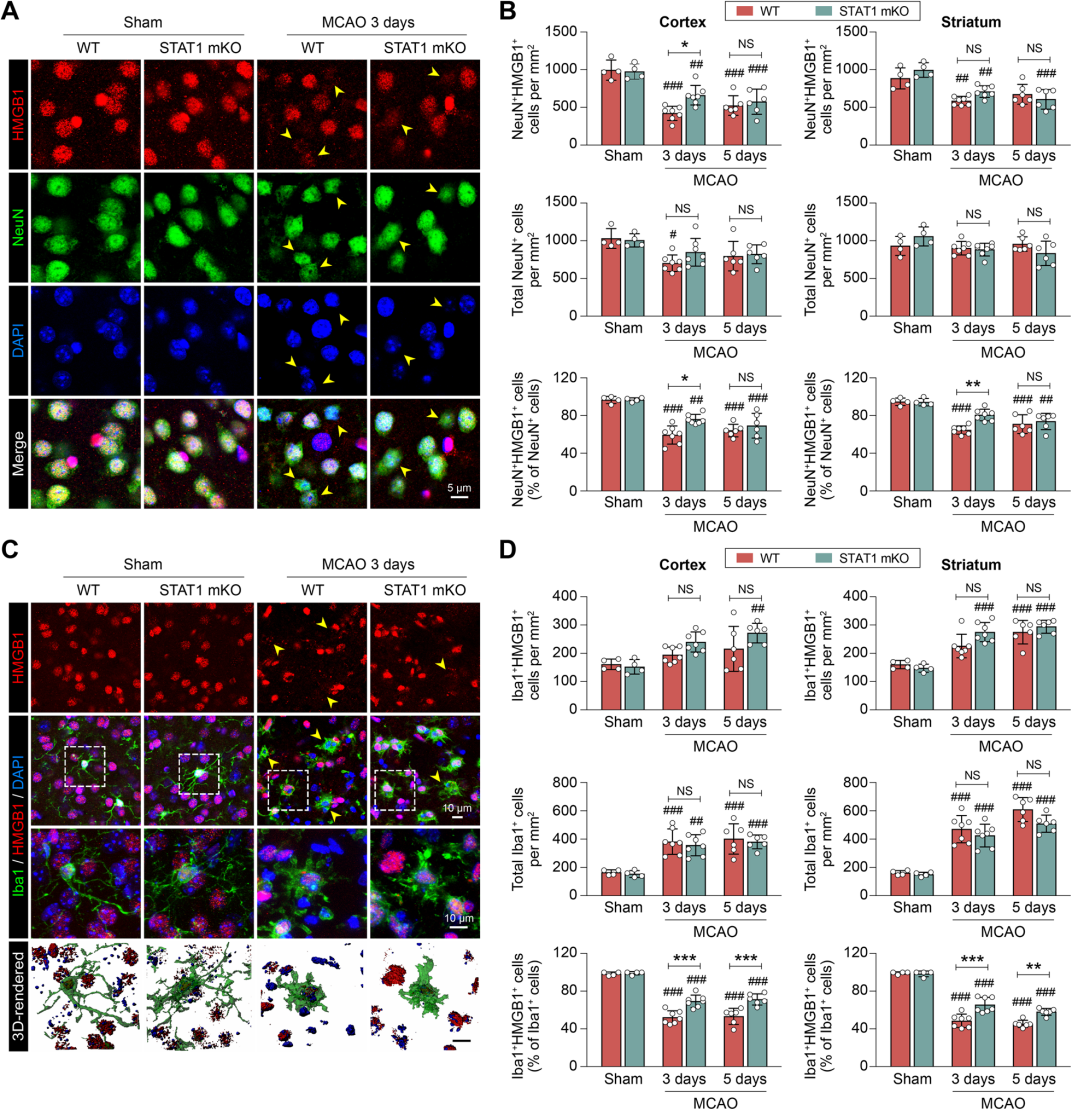
Microglia/macrophage-targeted STAT1 knockout alleviates subacute neurotoxic microglial/macrophage responses after ischemic stroke. STAT1 mKO mice and WT control mice were subjected to 1-h MCAO followed by 3 or 5 days of reperfusion. **A**, **B** Neuronal expression of HMGB1 was examined in the ipsilateral peri-infarct areas or corresponding areas in sham-operated brains by double-label immunostaining of NeuN and HMGB1. **A** Representative images taken from the cortex. *Arrowhead*: NeuN^+^ cells that are negative for HMGB1. **B** The numbers of NeuN^+^HMGB1^+^ cells were counted in the cortex and striatum 3 and 5 days after MCAO, and expressed as numbers per mm^2^ (*top panels*) or as percentages of total NeuN^+^ cells (*bottom panels*). **C**, **D** Microglia/macrophage expression of HMGB1 was examined by double-label immunostaining of Iba1 and HMGB1. **C** Representative images taken from the cortex. *Arrowhead*: Iba1^+^ cells that are negative for HMGB1. *Dashed rectangles*: Cells that are enlarged and 3D rendered in the 3rd and 4th rows, respectively. **C** The numbers of Iba1^+^HMGB1^+^ cells were counted in the cortex and striatum 3 and 5 days after MCAO, and expressed as numbers per mm^2^ (*top panels*) or as percentages of total Iba1^+^ cells (*bottom panels*). *n* = 4 (sham) or 6–7 (MCAO) mice/group. ^#^*p* < 0.05, ^##^*p* < 0.01, ^###^*p* < 0.001 MCAO vs. sham. *p < 0.05, **p < 0.001, ***p < 0.001 STAT1 mKO vs. WT. *NS* no significant difference

Despite lack of influence on acute neuronal death after brain ischemia, targeted deletion of STAT1 effectively reduced neurotoxic Mi/MΦ responses at the subacute injury stage. When we extended the examination of HMGB1 release from neurons to 3 and 5 days after MCAO (Fig. 4A), we found that the total number of NeuN^+^HMGB1^+^ cells remained significantly less than non-injured sham controls (Fig. 4B). Interestingly, the number of NeuN^+^HMGB1^+^ cells were higher in STAT1 mKO mice than WT mice 3 days after MCAO in both the peri-infarct cortex and striatum (Fig. 4B), possibly due to a decrease in neuronal stress signaling and retention of intracellular HMGB1 by neurons in STAT1 mKO mice. Since the STAT1 mKO mice had selective deletion of STAT1 in Mi/MΦ, we speculated that these neuroprotective effects of STAT1 mKO was secondary to reduced inflammatory signaling of Mi/MΦ. Therefore, we examined the expression of HMGB1 in Mi/MΦ as a marker for the healthiness of these cells. Through double-label immunostaining of Iba1 and HMGB1, we were able to quantify Mi/MΦ retention and release of HMGB1 at 3 and 5 days after MCAO (Fig. 4C). Compared to sham controls, MCAO increased the numbers of Iba1^+^ cells, reflecting infiltration of peripheral Iba1^+^ monocyte-derived macrophages (Fig. 4D). To compensate for such changes in the total number of Iba1^+^ cells, we quantified the percentages of Iba1^+^ cells that were immunopositive for HMGB1. The results showed that MCAO-induced release of HMGB1 from Iba1^+^ Mi/MΦ were significantly ameliorated in STAT1 mKO mice compared to WT mice in both the peri-infarct cortex and striatum, at both 3 days and 5 days after MCAO (Fig. 4D). In summary, these data suggest that STAT1-deficient Mi/MΦ have less propensity for DAMP inflammatory activity in the ischemic brain than their counterpart in WT mice at the subacute stage after ischemic stroke, which may lead to reduced secondary neuronal death.

### Deletion of STAT1 drives post-stroke Mi/MΦ toward an inflammation-resolving phenotype

**Fig. 5:**
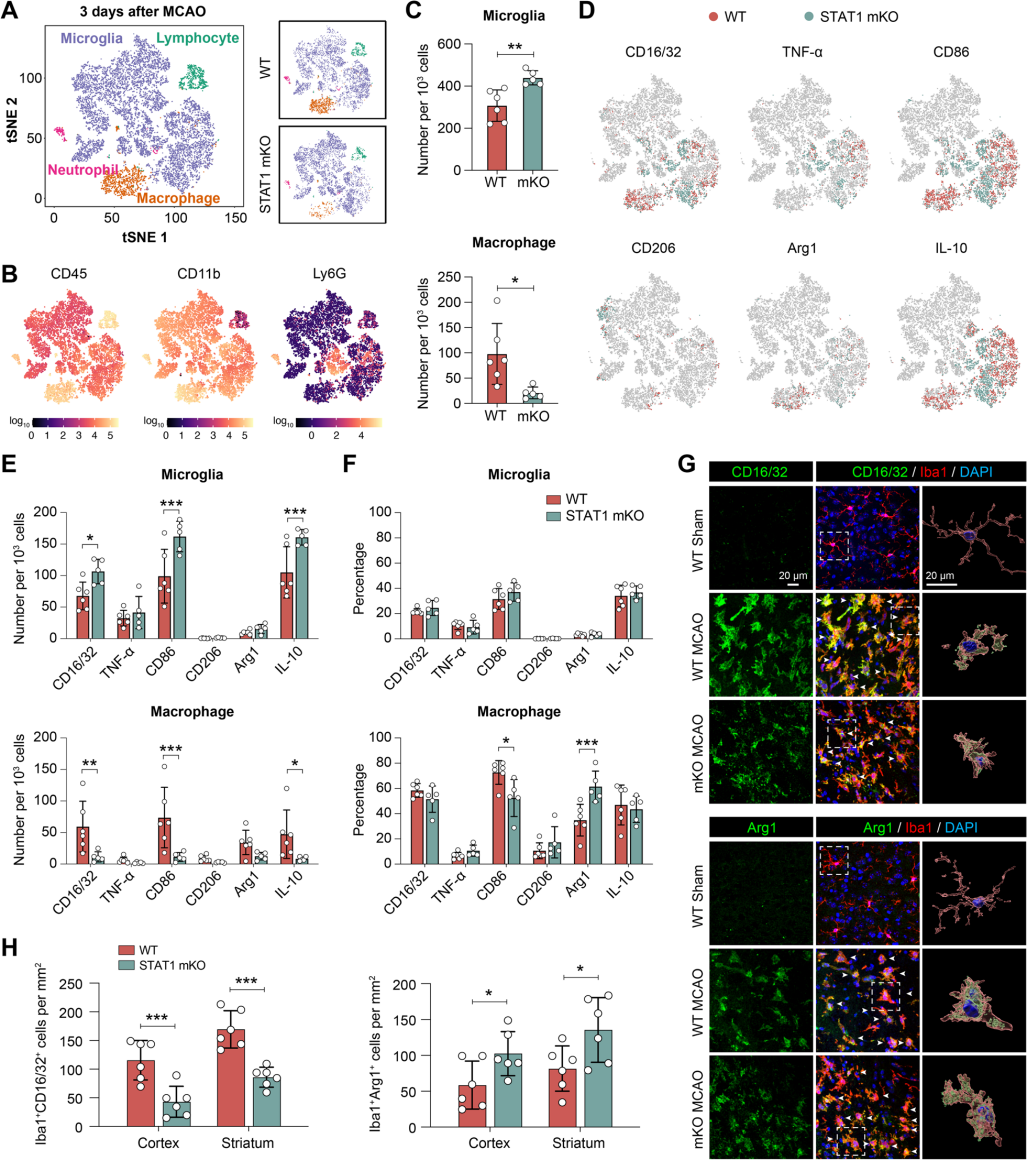
Loss of STAT1 ameliorates proinflammatory microglial/macrophage responses after ischemic stroke. **A**–**F** MCAO was induced in STAT1 mKO mice and WT mice. Three days after MCAO, flow cytometry was performed to assess the phenotype of microglia and macrophages in the ipsilesional brain hemisphere. **A** Representative t-SNE plots of 12,000 CD45^+^ cells pooled from one WT and one STAT1 mKO mouse brains. Each dot represents one cell. Color corresponds to cell type. **B** t-SNE plots showing the expression levels of prototypic cell markers in CD45^+^ cells that identified major cell types: microglia (CD11b^+^CD45^low^), macrophage (CD11b^+^CD45^high^), neutrophil (Ly6G^+^), and lymphocytes (CD11b^–^CD45^high^). Color represents fluorescence intensity on a logarithmic scale. **C** The numbers of microglia (Ly6G^–^CD11b^+^CD45^low^ cells) and macrophages (Ly6G^–^CD11b^+^CD45^high^ cells) in STAT1 mKO and WT mouse brains. **D** t-SNE plots of all microglia and macrophages demonstrate cells from WT and STAT1 mKO mice that were immunopositive for a panel of proinflammatory (CD16/32, TNF-α, CD86) and anti-inflammatory (CD206, arginase 1, IL-10) markers. **E**, **F** The numbers of microglia and macrophages positive for each marker were quantified after manual gating and expressed as cell number per 1000 single cells **E** or percentages of total microglia or macrophages (**F**). **G**, **H** The phenotype of microglia/macrophages was assessed 3 days after MCAO using double-label immunostaining of Iba1 and CD16/32 or arginase 1. Shown are representative images taken from the peri-infarct cortex **G** and summarized data on the numbers of double-immunopositive cells in the peri-infarct cortex and striatum (**H**). *n* = 5–6 mice/group. *p < 0.05, **p < 0.01, ***p < 0.001 STAT1 mKO vs. WT

We further assessed the functional phenotype of Mi/MΦ upon STAT1 deletion by examining a panel of markers indicative of their proinflammatory or inflammation-resolving functions at the subacute stage after MCAO. Using multicolor flow cytometry, we identified major subsets of immune cells in the post-MCAO brain (Fig. 5A) based on their expression of prototypic markers (Fig. 5B). The macrophage population (CD11b^+^CD45^high^ cells) was smaller in STAT1 mKO mice than WT mice 3 days after MCAO (Fig. 5C), likely reflecting a general reduction of inflammatory responses in the brain. As a result, the number of microglia (CD11b^+^CD45^low^ cells) per 1000 single cells examined was relatively larger in STAT1 mKO mice than WT mice (Fig. 5C). To account for the variation in total numbers of microglia and macrophages, we quantified the cells expressing a specific phenotypic marker both as the number per 1000 single cells and as a percentage of total microglia or macrophage numbers. This approach enabled us to accurately assess and compare the presence of cells with the desired phenotypic characteristics, irrespective of the differences in overall microglia and macrophage counts. A panel of 6 phenotypic markers was examined, including the proinflammatory markers CD16/32, TNF-α, CD86, and markers indicative of inflammation-resolving activities CD206, Arg1, and IL-10 (Fig. 5D). The percentage of microglia expressing all 6 markers were similar between STAT1 mKO mice and WT mice (Fig. 5E, F), suggesting negligible changes in microglia phenotype induced by STAT1 mKO. In contrast, macrophages in STAT1 mKO mice exhibited a substantial alleviation in their proinflammatory profile. The percentage of proinflammatory macrophages expressing CD86 was significantly reduced, whereas the inflammation-resolving macrophages expressing Arg1 was markedly elevated, in STAT1 mKO mice compared to WT mice 3 days after MCAO (Fig. 5E, F).

We further verified the findings from flow cytometry using immunostaining of Iba1, double-labeled with the proinflammatory phenotypic marker CD16/32 and the inflammation-resolving marker Arg1 (Fig. 5G). Consistent with flow cytometry findings, the number of CD16/32^+^ Mi/MΦ was significantly less in STAT1 mKO mice than WT mice, and the number of Arg1^+^ Mi/MΦ were increased in STAT1 mKO mice, in both the peri-infarct cortex and striatum 3 days after MCAO (Fig. 5H). In summary, these data suggest that STAT1 mKO potently shifts the phenotype of Mi/MΦ toward an inflammation-resolving phenotype after ischemic stroke.

### Targeted knockout of STAT1 Mi/MΦ mitigates brain inflammation after ischemic stroke

**Fig. 6:**
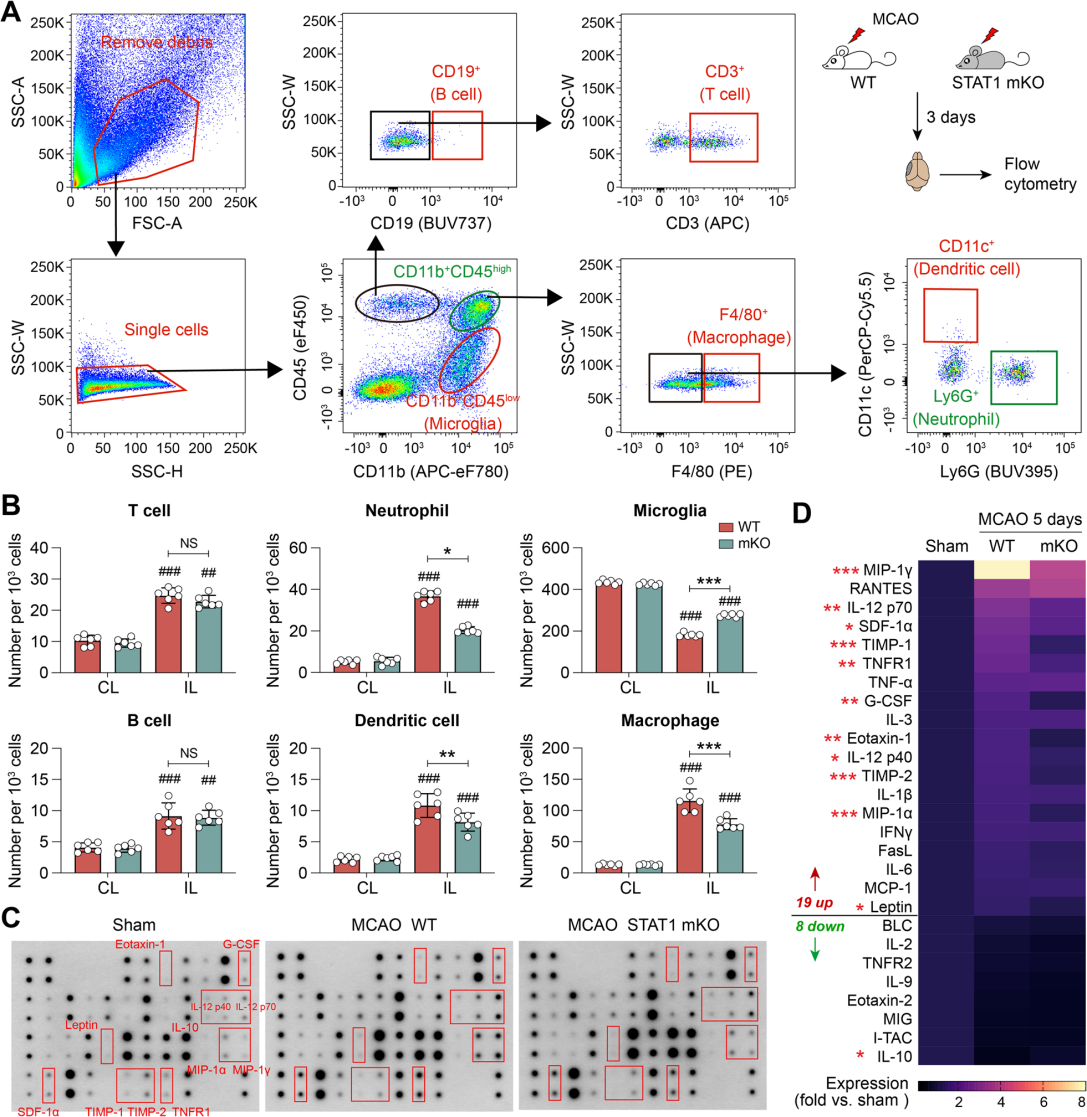
Selective deletion of STAT1 in microglia/macrophages mitigates brain inflammation after ischemic stroke. STAT1 mKO mice and WT mice were subjected to 1-h MCAO followed by 3 or 5 days of reperfusion. **A**, **B** Flow cytometry was performed 3 days after MCAO to assess the infiltration of peripheral immune cells into the post-MCAO brain. **A** Gating strategy for various immune cells in the brain. **B** The numbers of various immune cells in the ipsilesional (IL) and non-injured contralesional (CL) brain hemispheres. **C**, **D** A panel of 40 inflammatory cytokines was measured in the ipsilesional brain hemisphere 5 days after MCAO using an antibody array. **C** Representative blots with significantly altered markers labeled. **D** Heat map showing the mean expression levels of 17 markers that were significantly different among groups. Sham controls were pooled from both WT and STAT1 mKO mice, between which there was no significant difference. *n* = 6 mice/group. ^##^*p* < 0.01, ^###^*p* < 0.001 IL vs. CL. *p < 0.05, **p < 0.01, ***p < 0.001 STAT1 mKO vs. WT after MCAO. *NS* no significant difference

Mi/MΦ are important mediators of inflammatory responses in the post-stroke brain, which greatly influences stroke outcomes [2, 3]. Next, we examined whether alteration of Mi/MΦ phenotype by STAT1 deletion led to secondary changes in the brain inflammation profiles at the subacute stage after stroke. We first determined the inflammation burden of the post-stroke brain by measuring the presence of infiltrating blood immune cells 3 days after MCAO (Fig. 6A). Flow cytometry analysis showed that MCAO-induced peripheral immune cell infiltration into the brain was markedly reduced 3 days after MCAO in STAT1 mKO mice compared to WT mice (Fig. 6B). There were significantly less neutrophils, dendritic cells, and CD11b^+^CD45^high^ macrophages and activated microglia in the ipsilateral hemisphere of ischemic STAT1 mKO mice, whereas the numbers of T cells and B cells were comparable between ischemic STAT1 mKO mice and WT mice (Fig. 6B). As a result, the relative portion of CD11b^+^CD45^low^ cells (mostly quiescent microglia) per 1000 single cells examined was increased in the ischemic hemisphere of STAT1 mKO mice compared to WT mice (Fig. 6B). In parallel, we determined the content of 40 inflammatory cytokines in the brain 5 days after MCAO using an antibody array (Fig. 6C). Compared to the baseline inflammation level in sham controls, MCAO elevated 19 inflammation markers, including the proinflammatory cytokines IL-6, IL-12, and the chemokines attracting migrating immune cells MIP-1α/CCL3, and MIP-1γ/CCL9 (Fig. 6D). The expression of 11 out of these 19 markers which may exacerbate brain injury by potentiating neuroinflammation was significantly reduced in STAT1 mKO mice compared to WT mice 5 days after MCAO (Fig. 6D). Interestingly, the expression of the anti-inflammatory cytokine IL-10, which was downregulated by MCAO, was relatively increased in ischemic STAT1 mKO mice compared to ischemic WT mice (Fig. 6D). These data consistently demonstrated mitigation of inflammation in the post-stroke brain by Mi/MΦ-targeted STAT1 deletion.

### STAT1 mKO is sufficient to improve long-term functional recovery after stroke in both male and female mice

**Fig. 7:**
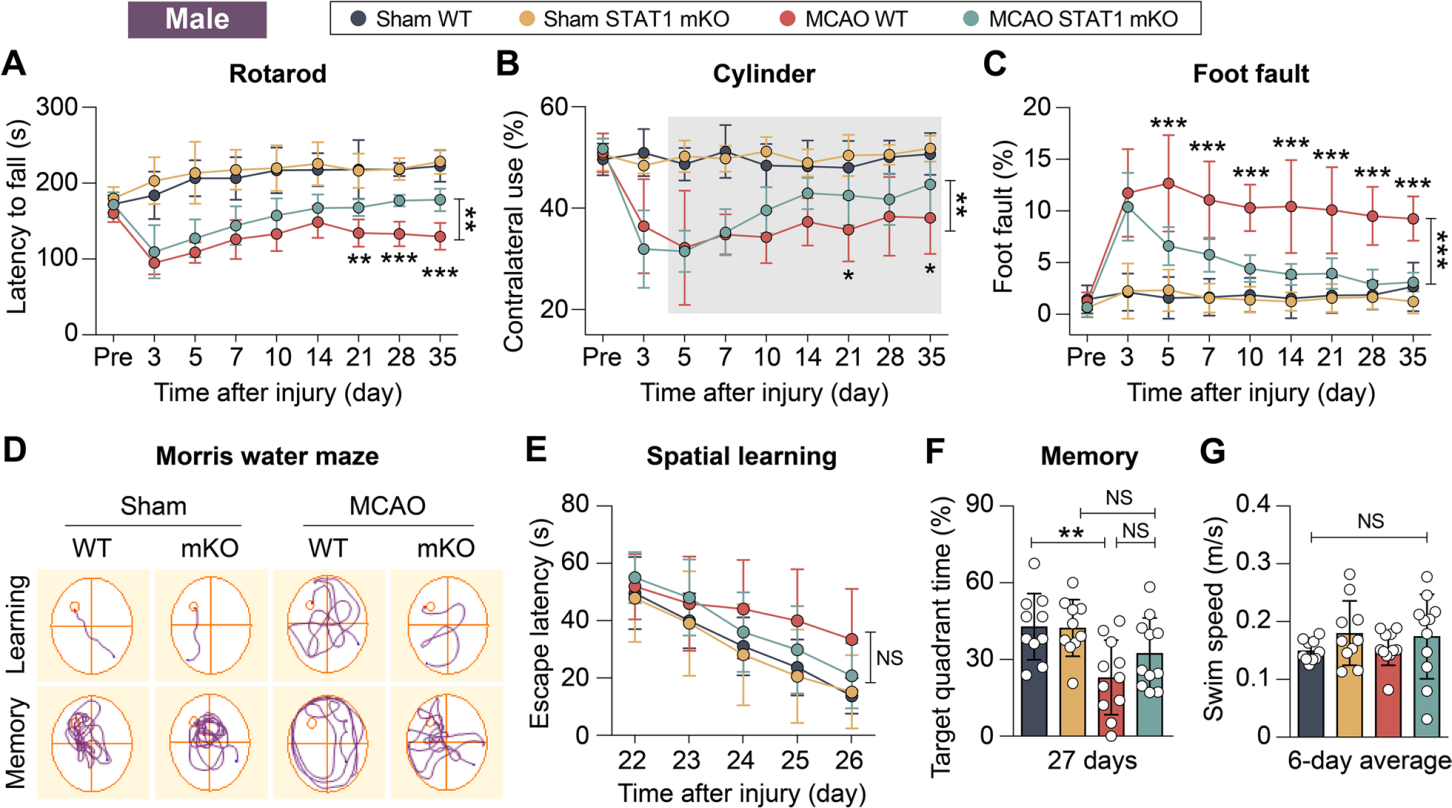
Microglia/macrophage-targeted STAT1 knockout improves long-term neurological functions in male mice after ischemic stroke. Adult male STAT1 mKO mice and WT mice were subjected to 1-h MCAO or sham operation. **A**–**C** Sensorimotor functions were assessed before (Pre) and up to 35 days after injury by the rotarod test (**A**), cylinder test (**B**), and foot fault test (**C**). **D**–**G** Cognitive functions were assessed by the Morris water maze 22–27 days after injury. **D** Representative swim paths in the learning and memory phases of the Morris water maze test. **E** Spatial learning assessed 22–26 days after injury demonstrated no difference among all groups. **F** Spatial memory assessed 27 days after injury. **G** All groups had similar swim speeds, indicating comparable gross locomotor functions. *n* = 10–11 mice/group. *p < 0.05, **p < 0.01, ***p < 0.001 mKO MCAO vs. WT MCAO by two-way repeated measures ANOVA (bracket in **A**, **B**, **C**, **E**) or one-way ANOVA (individual timepoint in **A**, **B**, **C**, **E**; **F** and **G**). In **B**, data on days 5–35 were analyzed. *NS* no significant difference

To determine the extent to which the alterations in Mi/MΦ phenotype resulting from STAT1 mKO impact the overall outcome following ischemic stroke, the recovery of neurological functions was evaluated in STAT1 mKO mice over a 35-day period. We employed a panel of three behavioral tests, namely, the rotarod test, cylinder test, and foot fault test, to assess the sensorimotor functions of adult male mice (Fig. 7A–C). The results obtained from all three tests consistently demonstrated significant sensorimotor deficits in the mice following MCAO when compared to sham-operated mice (Fig. 7A–C). Importantly, STAT1 mKO mice demonstrated significantly better sensorimotor functions in all three tests (Fig. 7A–C; *p* < 0.01 or *p* < 0.001). We also carried out the Morris water maze test to assess the animals’ spatial learning and spatial memory at 22–26 days after MCAO (Fig. 7D), but STAT1 mKO did not cause significant difference compared to WT mice in either the learning phase or the memory phase of this test (Fig. 7E–G). Collectively, these data suggest that by promoting inflammation-resolving Mi/MΦ responses, STAT1 mKO is effective in inducing long-term improvements in functional recovery following MCAO. These findings highlight the potential of STAT1 as a promising therapeutic target for the treatment of ischemic stroke.

**Fig. 8:**
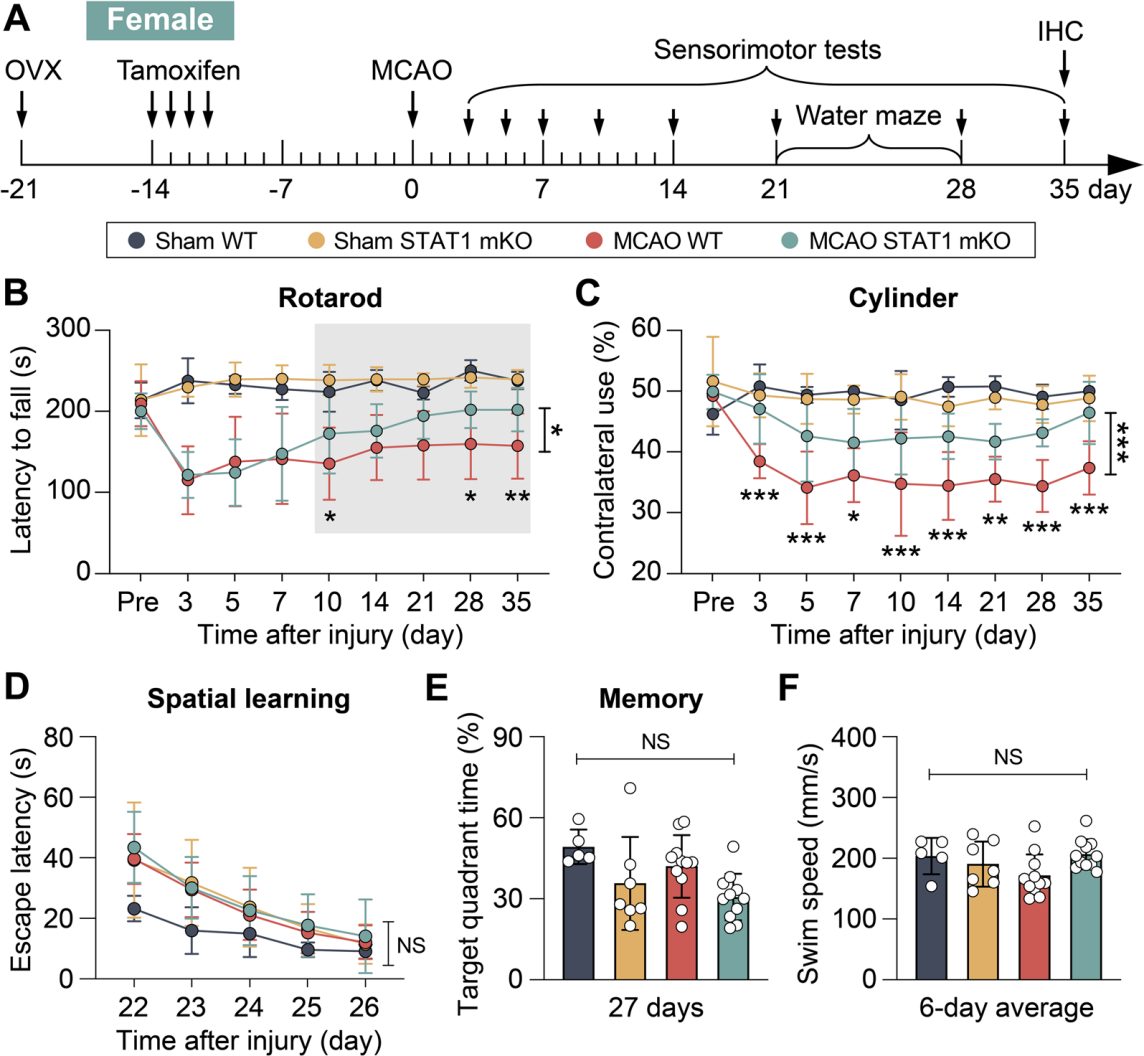
Microglia/macrophage-targeted STAT1 knockout ameliorates long-term neurological deficits after ischemic stroke in female mice.** A** Experimental timeline. Female STAT1 mKO mice and WT mice were subjected to ovariectomy (OVX). One week after ovariectomy, mice received intraperitoneal injections of tamoxifen (75 mg/kg daily for 4 days) to induce the deletion of STAT1 in microglia/macrophages. Mice were subjected to 1-h MCAO or sham operation 3 weeks after ovariectomy. **B**, **C** Sensorimotor deficits were examined in ovariectomized female STAT1 mKO and WT mice up to 35 days after injury by the rotarod test **B** and cylinder test (**C**). **D**–**F** Spatial learning and memory were assessed in female mice 22–27 days after injury by the Morris water maze. *n* = 5–7 mice per sham group. *n* = 11 mice per MCAO group. *p < 0.05, **p < 0.01, ***p < 0.001 mKO MCAO vs. WT MCAO by two-way repeated measures ANOVA (bracket in **B**–**D**) or one-way ANOVA (individual timepoint in **B**–**F**). In **B**, data on days 10–35 were analyzed. NS, no significant difference

Sex differences in the outcome after stroke and responsiveness to therapeutics are well-noted. To enhance the translatability of stroke therapeutics, it is crucial to conduct testing of interventions that have been proven effective in male subjects on female subjects as well [32]. To this end, we tested whether STAT1 mKO had similar beneficial effect in improving long-term outcome after stroke in female mice. To minimize any potential confounding effects associated with gonadal hormones and to better reflect the conditions observed in aged female stroke patients [33–35], we performed ovariectomy prior to MCAO (Fig. 8A). Ovariectomy leads to the depletion of endogenous estrogen levels and models surgical menopause to replicate the hormonal milieu of postmenopausal women to a certain extent. Consistent with the observations in male mice, female STAT1 mKO had better sensorimotor function than age-matched female WT mice for at least 35 days after MCAO, as tested by the rotarod (Fig. 8B) and cylinder (Fig. 8C) tests. Genotype did not cause differences in the animals’ performance in the Morris water maze test at 22–27 days after MCAO (Fig. 8D–F). These data suggest that the long-term beneficial effect of STAT1 mKO toward improving post-stroke sensorimotor functions is not sex-dependent.

### Selective deletion of STAT1 in Mi/MΦ promotes the integrity white matter after ischemic stroke

**Fig. 9:**
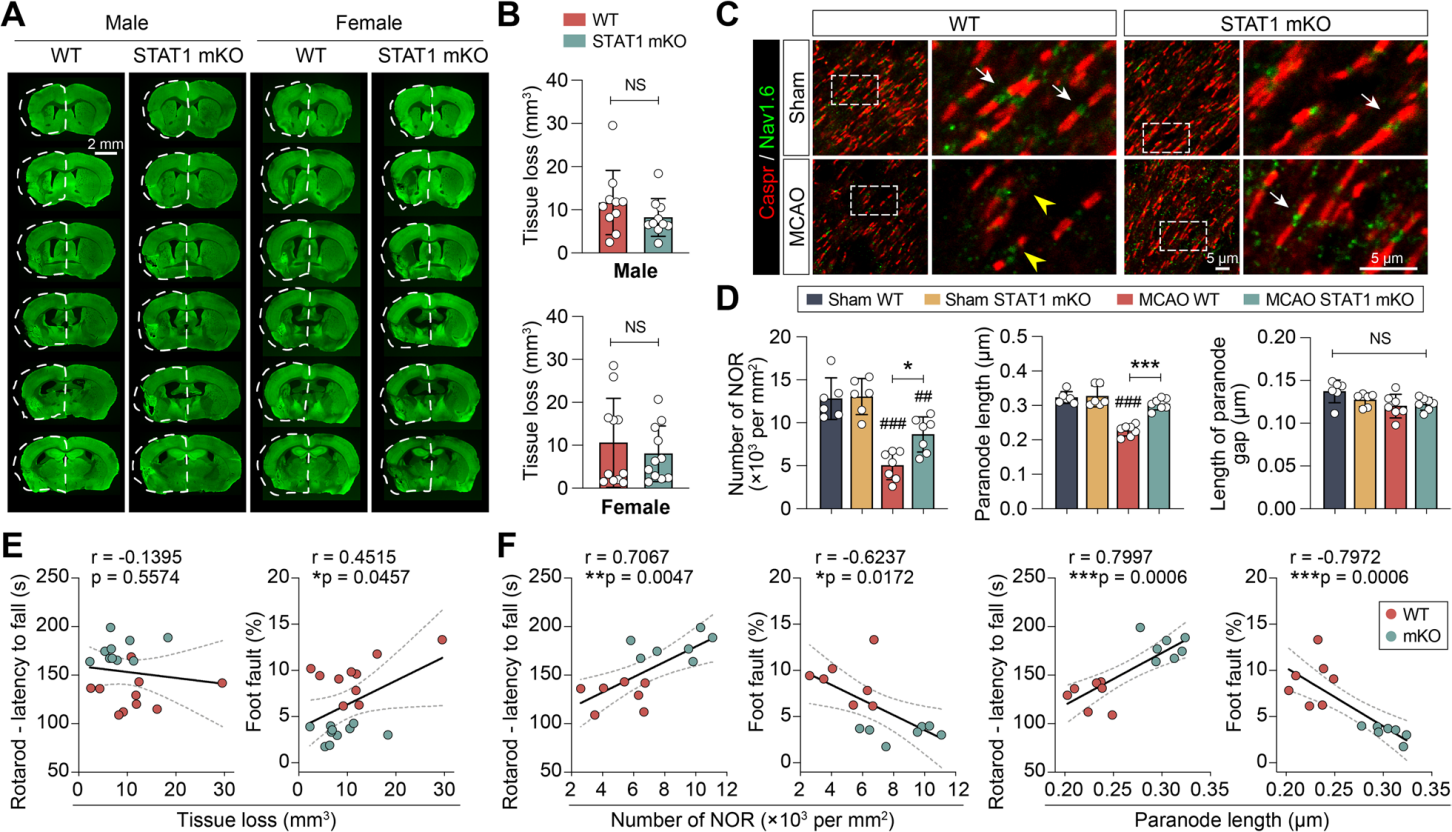
Microglia/macrophage-targeted STAT1 knockout improves long-term white matter integrity after ischemic stroke. **A**, **B** Chronic neuronal tissue loss was assessed in male and ovariectomized female STAT1 mKO mice and WT mice 35 days after MCAO on coronal brain sections immunostained for MAP2 (*green*). *Dash line* shows the relative area of the contralesional hemisphere to illustrate ipsilesional tissue atrophy. *n* = 10–11 mice/group. **C** The integrity of nodes of Ranvier (NORs) was assessed by double-label immunostaining of Caspr and Nav1.6 in the peri-lesion external capsule 35 days after MCAO or sham operation. *Arrow*: intact NOR. *Arrowhead*: damaged NOR. *Rectangle*: the region enlarged in the right columns. **D** Summarized data on the numbers of NORs, paranode lengths, and lengths of paranode gaps. *n* = 6–7 mice/group. ^##^*p* < 0.01, ^###^*p* < 0.001 vs. sham. *p < 0.05, ***p < 0.001 STAT1 mKO vs. WT. NS, no significant difference. **E**, **F** Pearson correlation between the animals’ performance in the rotarod test and foot fault test, and various parameters representing gray matter **E** and white mater **F** integrity 35 days after MCAO. *Dashed lines*: 95% confidence intervals

We observed that ischemic brain tissue loss at the acute stage after MCAO was not different between STAT1 mKO and WT mice (Fig. 3B, C). However, given the long-lasting functional improvement elicited by STAT1 mKO after MCAO, we sought to determine if STAT1 mKO affected the long-term integrity of the post-stroke brain. Chronic tissue loss in the gray matter measured on coronal brain sections immunostained with the neuronal marker MAP2 at 35 days after MCAO (Fig. 9A) was not significantly different between STAT1 mKO mice and WT mice, regardless of their sex (Fig. 9B), consistent with observations at more acute stages following MCAO (Fig. 3B, C). However, STAT1 mKO markedly improved the integrity of white matter. We labeled the nodes of Ranvier (NORs) in the ipsilateral external capsule—a white matter-enriched region—with contactin-associated protein (Caspr) and sodium channel Nav1.6 [16]. Caspr represents an axonal membrane protein found at the nodes, while Nav1.6 is predominantly located at the paranodes (Fig. 9C). An intact NOR was defined as the presence of a gap positive for Nav1.6 between a pair of paranodal Caspr staining. We observed significant loss of morphologically intact NORs in the external capsule at 35 days after MCAO, with a concomitant reduction in the length of paranodal Caspr immunosignal of individual NORs (Fig. 9C, D). Such NOR damage induced by MCAO was effectively ameliorated in STAT1 mKO mice compared to WT mice at 35 days after MCAO (Fig. 9C, D), suggesting improved white matter integrity in the STAT1 mKO mice. The lengths of the NORs (paranodal gaps) were not different among all groups (Fig. 9D).

To test whether the improved white matter integrity underlies better sensorimotor functions in the STAT1 mKO mice, we performed Pearson correlation analyses on the same cohort of mice subjected to both neurobehavioral tests and immunostaining. While the gray matter tissue loss at 35 days after MCAO did not demonstrate strong correlation with the animals’ sensorimotor deficits detected by the rotarod test and foot fault test (Fig. 9E), the animals’ performance in both behavioral tests was significantly correlated with the parameters for white matter integrity (number of NORs and paranode length; Fig. 9F). In conclusion, these data suggest that STAT1 mKO-elicited long-term functional improvement after MCAO was attributable, at least partially, to the rescued white matter integrity.

## Discussion

In this study, we utilized a conditional knockout mouse model to delineate the role of STAT1 in modulating the function of Mi/MΦ following an ischemic stroke. Our findings provide compelling evidence that STAT1 plays a significant role in promoting proinflammatory Mi/MΦ responses. Importantly, in vivo selective deletion of STAT1 drives Mi/MΦ toward an inflammation-resolving phenotype, resulting in mitigation of neuroinflammation and improvement of long-term stroke outcomes.

STAT1 is a transcription factor that is potently activated in canonical interferon signaling, and is defined as an important mediator of macrophage proinflammatory M1 polarization [36]. Independent of its role downstream of interferon gamma, STAT1 also promotes apoptosis through regulating caspases [37, 38]. While the pro-apoptotic role of STAT1 had been examined in the context of ischemic stroke in previous studies [9, 39, 40], its proinflammatory function had so far only been implicated in an indirect and correlative way in existing studies. Reduced activity of STAT1 was found by several studies to accompany less neuroinflammation upon treatment of anti-inflammatory drugs [41, 42]; however, a direct role of STAT1 in regulating inflammation, especially through modulating the phenotype of Mi/MΦ, is unknown. Our study addressed several crucial questions that were previously unanswered. First, we revealed the temporal profile of STAT1 expression in different types of brain cells during the acute to subacute stages following brain ischemia and reperfusion. Second, by utilizing Mi/MΦ-targeted conditional knockout of STAT1, we were able to specifically investigate the role of STAT1 in Mi/MΦ, separate from its effects in other cell types, such as neurons. While the deletion of STAT1 in Mi/MΦ did not directly impact acute neuronal death, it resulted in decreased proinflammatory and neurotoxic Mi/MΦ responses. Third, our study established a direct role for Mi/MΦ STAT1 in determining long-term stroke outcomes. Although compensatory changes in Mi/MΦ or other cell types may occur, they are secondary to the primary effects of Mi/MΦ STAT1 deletion.

As a transcription factor, STAT1 has a lot of target genes. In our study, we observed changes in several pre-defined phenotype markers upon STAT1 deletion in Mi/MΦ, one of which is Arg1. Arg1 metabolizes L-arginine into urea and ornithine and is a countermeasure to iNOS, which produces nitric oxide from arginine [43]. Arg1 is both a prototypic marker and a strong inducer of M2 macrophages [43]. The functional implication of Arg1 is twofold: first, by competing with iNOS for substrates, Arg1 has potent anti-inflammatory effect; second, Arg1 was previously reported to enhance phagocytic function of Mi/MΦ after ischemic stroke [22, 44]. It is, therefore, an important question for future studies to assess whether STAT1 deletion promotes phagocytic clearance of debris or dead cells by Mi/MΦ. The mechanism underlying the increase in Arg1 upon STAT1 knockout is not yet fully understood. Transcription of Arg1 is prominently induced by IL-4, which requires the direct binding of both STAT6 and cAMP-response element binding protein (CREB)-binding protein (CBP) to a special DNA response element in the Arg1 promoter [45, 46]. We are currently testing the possibility that STAT1 suppresses Arg1 expression by inhibiting the binding of CBP to the Arg1 promoter. On the other hand, proinflammatory (“M1”-like) and inflammation-resolving (“M2”-like) markers were largely derived from in vitro polarization studies and may not be as informative about the activation and functional states of Mi/MΦ in vivo. In this regard, our ongoing studies are also exploring changes in gene expression of STAT1-null Mi/MΦ using unbiased RNA-seq at the whole transcriptome level. This comprehensive approach will enable us to gain deeper insights into the functional states of Mi/MΦ and downstream targets of STAT1, going beyond the pre-defined phenotypic markers examined in the current study. By analyzing the entire transcriptome, we aim to identify novel targets and pathways that are influenced by STAT1 deletion in Mi/MΦ. It is important to acknowledge that our current method of standard enzymatic digestion of brain tissues at 37 °C to prepare cell suspensions for RNA-seq could potentially induce ex vivo transcriptional changes in microglia [47]. However, we took measures to mitigate the impact of these artifacts by subjecting all groups to identical cell isolation procedures for a fair comparison. Future studies should consider optimizing the cell isolation protocols, e.g., by adding transcriptional and translational inhibitors or using a mechanical dissociation protocol conducted at low temperatures [47] to minimizing the activation of microglia ex vivo.

One limitation of our study is that we did not clearly separate microglia from macrophages in most of our assessment parameters due to the lack of definitive markers that can reliably distinguish between these two cell types. Traditionally, microglia have been considered as CD45^low^, while macrophages are CD45^high^. However, activated microglia can upregulate CD45 expression and become indistinguishable from macrophages in terms of CD45 expression. In addition, recently identified markers, such as Tmem119, which were thought to be "microglia-specific," may have altered expression under disease conditions [48], raising concerns about their reliability as markers for microglia in the context of ischemic injury. In our study, we utilized Mi/MΦ-targeted STAT1 knockout mice generated under the *Cx3cr1* promoter, which is strongly expressed by both microglia and macrophages, including those macrophages residing at the CNS borders (i.e., meningeal, perivascular and choroid-plexus macrophages) [49]. Using these mice, we found STAT1 in both microglia and macrophages could be important. Future studies can employ strategies to more definitively separate microglia and macrophages to determine if microglia and macrophage STAT1 have redundant functions or if one dominates over the other in driving the phenotypic changes observed. For example, a tamoxifen pulse knockout strategy employed in our recent study [50] could be used to restrict the deletion of STAT1 to only occur in long-living cells, such as microglia. Recent advancements in identifying new markers may also lead to the development of Cre strains with improved targeting specificity, particularly in distinguishing microglia from CNS border-associated macrophages [48]. Notably, the Cx3cr1^CreER^ mice used in this study did not target all monocyte-derived macrophages due to the existence of the CX3CR1^−^Ly6C^hi^ monocyte subset [51]. Therefore, in our STAT1 mKO mice, STAT1 deletion occurred in most of the microglia but only part of the macrophages in the brain. To further reveal the role of macrophage STAT1, future studies utilizing monocyte-targeting tools (e.g., the recently developed CCR2^CreER^ mice [52]) are warranted. Another limitation of the Cx3cr1^CreER^ mice used in the current study is that these mice lack one copy of the full-length endogenous *Cx3cr1* allele [11]. To compensate for the potential impact of CX3CR1 haploinsufficiency on the phenotype of Mi/MΦ, we used Cx3cr1^CreER^ hemizygous mice as WT controls to the STAT1 mKO mice, so that both groups of mice had identical *Cx3cr1* alleles.

We found that the deletion of STAT1 in Mi/MΦ resulted in less brain inflammation at the subacute stage after ischemic stroke, manifested by less infiltration of blood-borne immune cells and reduced content of cytokines in the post-stroke brain. However, it is important to note that immune cells and cytokines may also play a beneficial role in the chronic stage of stroke by aiding in tissue repair and remodeling. While the deletion of STAT1 in Mi/MΦ did not affect chronic neuronal tissue loss at 35 days after MCAO, it had a significant positive impact on white matter integrity. The improvement in white matter integrity is believed to contribute to the observed enhancement in sensorimotor functions. We observed improvements in neurological functions in both male and female STAT1 mKO mice, suggesting that the therapeutic targeting of STAT1 may be effective regardless of sex. It should be noted that in our study, ovariectomized female mice were used to partially mimic the systemic conditions of aged women, who make up the majority of stroke patients. However, it is worth considering that there may be other hormone-independent mechanisms that contribute to sex differences in treatment efficacy. Considering the known differences in microglia physiology during aging [53, 54], future studies should aim to test the conclusions of our research in aged subjects to provide valuable insights for potential therapeutic interventions in elderly stroke patients.

The identification of therapeutic targets for stroke treatment remains an urgent need. In our current study, we employed genetically engineered mice and demonstrated that STAT1 is a promising target for reducing inflammation and secondary ischemic injury by modulating Mi/MΦ phenotype. Future studies are warranted to investigate the translatability of the conclusions drawn from the present study to clinical stroke treatment. To this end, STAT1 inhibitors are promising agents to be tested for their therapeutic efficacy against ischemic stroke, e.g., the purine analog fludarabine. Fludarabine is approved by the FDA to treat hematologic malignancies for its ability to inhibit DNA synthesis [55]. It can also specifically deplete STAT1 mRNA and protein but not other STATs [56], thereby exerting anti-inflammatory and immunosuppressive effects. As a nucleoside analog, fludarabine can easily cross the BBB, and oral administration of this drug has similar tolerability profile and clinical efficacy compared to the intravenous route [57, 58]. We recently reported that systemically administered fludarabine exert potent anti-inflammatory effects in a mouse model of traumatic brain injury [19]. We are currently conducting investigations to assess the therapeutic potential of this drug for the treatment of ischemic stroke.

## Conclusions

STAT1 plays a significant role in promoting proinflammatory responses of Mi/MΦ following ischemic stroke. By selectively deleting STAT1 in Mi/MΦ, the post-stroke neuroinflammation can be effectively mitigated, leading to improved long-term functional recovery. These findings highlight the potential of targeting STAT1 as a promising therapeutic approach for stroke treatment. Such intervention has the potential to enhance beneficial Mi/MΦ responses and ultimately improve long-term outcomes in stroke patients.

## Supplementary Information

**Additional file 1: Table S1.** Key resources.

**Additional file 2: Table S2.** List of the 2183 differentially expressed genes (DEGs) induced by MCAO in microglia compared to sham controls (related to Fig. 1). Genes are ranked according to fold changes (MCAO vs. sham) from smallest to largest.

## Data Availability

RNA sequencing data are deposited in the Gene Expression Omnibus database at the National Center for Biotechnology Information (GSE141972 and GSE234865). Other data sets used and/or analyzed in this study are available from the corresponding author upon reasonable request.

## Abbreviations

Arg1: Arginase 1
Caspr: Contactin-associated protein
DAMP: Damage-associated molecular pattern
DEG: Differentially expressed gene
FACS: Fluorescence-activated cell sorting
FDR: False discovery rate
HMGB1: High mobility group box 1
IL: Interleukin
iNOS: Inducible nitric oxide synthase
IPA: Ingenuity pathway analysis
MAP2: Microtubule-associated protein 2
MCA: Middle cerebral artery
MCAO: Middle cerebral artery occlusion
Mi/MΦ: Microglia and macrophages
mKO: Microglia/macrophage-specific knockout
NOR: Node of Ranvier
PCR: Polymerase chain reaction
rCBF: Regional cerebral blood flow
RNA-seq: RNA sequencing
STAT: Signal transducer and activator of transduction
TNF-α: Tumor necrosis factor alpha
WT: Wild type

## References

[1] Shi L, Rocha M, Leak RK, Zhao J, Bhatia TN, Mu H (2018). A new era for stroke therapy: Integrating neurovascular protection with optimal reperfusion. J Cereb Blood Flow Metab.

[2] An C, Shi Y, Li P, Hu X, Gan Y, Stetler RA (2014). Molecular dialogs between the ischemic brain and the peripheral immune system: dualistic roles in injury and repair. Prog Neurobiol.

[3] Jiang X, Andjelkovic AV, Zhu L, Yang T, Bennett MVL, Chen J (2018). Blood-brain barrier dysfunction and recovery after ischemic stroke. Prog Neurobiol.

[4] Hu X, Leak RK, Shi Y, Suenaga J, Gao Y, Zheng P (2015). Microglial and macrophage polarization-new prospects for brain repair. Nat Rev Neurol.

[5] Hu X, Li P, Guo Y, Wang H, Leak RK, Chen S (2012). Microglia/macrophage polarization dynamics reveal novel mechanism of injury expansion after focal cerebral ischemia. Stroke.

[6] Han W, Song Y, Rocha M, Shi Y (2023). Ischemic brain edema: Emerging cellular mechanisms and therapeutic approaches. Neurobiol Dis.

[7] Ivashkiv LB, Donlin LT (2014). Regulation of type I interferon responses. Nat Rev Immunol.

[8] Miklossy G, Hilliard TS, Turkson J (2013). Therapeutic modulators of STAT signalling for human diseases. Nat Rev Drug Discov.

[9] Takagi Y, Harada J, Chiarugi A, Moskowitz MA (2002). STAT1 is activated in neurons after ischemia and contributes to ischemic brain injury. J Cereb Blood Flow Metab.

[10] Klover PJ, Muller WJ, Robinson GW, Pfeiffer RM, Yamaji D, Hennighausen L (2010). Loss of STAT1 from mouse mammary epithelium results in an increased Neu-induced tumor burden. Neoplasia.

[11] Parkhurst CN, Yang G, Ninan I, Savas JN, Yates JR, Lafaille JJ (2013). Microglia promote learning-dependent synapse formation through brain-derived neurotrophic factor. Cell.

[12] Kilkenny C, Browne WJ, Cuthill IC, Emerson M, Altman DG (2010). Improving bioscience research reporting: the ARRIVE guidelines for reporting animal research. PLoS Biol.

[13] Fisher M, Feuerstein G, Howells DW, Hurn PD, Kent TA, Savitz SI (2009). Update of the stroke therapy academic industry roundtable preclinical recommendations. Stroke.

[14] Shi Y, Zhang L, Pu H, Mao L, Hu X, Jiang X (2016). Rapid endothelial cytoskeletal reorganization enables early blood-brain barrier disruption and long-term ischaemic reperfusion brain injury. Nat Commun.

[15] Liu Y, Li S, Wang R, Pu H, Zhao Y, Ye Q (2021). Inhibition of TGFbeta-activated kinase 1 promotes inflammation-resolving microglial/macrophage responses and recovery after stroke in ovariectomized female mice. Neurobiol Dis.

[16] Wang R, Pu H, Ye Q, Jiang M, Chen J, Zhao J (2020). Transforming growth factor beta-activated kinase 1-dependent microglial and macrophage responses aggravate long-term outcomes after ischemic stroke. Stroke.

[17] Shi Y, Jiang X, Zhang L, Pu H, Hu X, Zhang W (2017). Endothelium-targeted overexpression of heat shock protein 27 ameliorates blood-brain barrier disruption after ischemic brain injury. Proc Natl Acad Sci U S A.

[18] Wang R, Liu Y, Ye Q, Hassan SH, Zhao J, Li S (2020). RNA sequencing reveals novel macrophage transcriptome favoring neurovascular plasticity after ischemic stroke. J Cereb Blood Flow Metab.

[19] Zhao Y, Ma C, Chen C, Li S, Wang Y, Yang T (2022). STAT1 contributes to microglial/macrophage inflammation and neurological dysfunction in a mouse model of traumatic brain injury. J Neurosci.

[20] Linderman GC, Rachh M, Hoskins JG, Steinerberger S, Kluger Y (2019). Fast interpolation-based t-SNE for improved visualization of single-cell RNA-seq data. Nat Methods.

[21] R Core Team. R: A language and environment for statistical computing. Vienna: R Foundation for Statistical Computing; 2020.

[22] Zhang W, Zhao J, Wang R, Jiang M, Ye Q, Smith AD (2019). Macrophages reprogram after ischemic stroke and promote efferocytosis and inflammation resolution in the mouse brain. CNS Neurosci Ther.

[23] Kallio MA, Tuimala JT, Hupponen T, Klemela P, Gentile M, Scheinin I (2011). Chipster: user-friendly analysis software for microarray and other high-throughput data. BMC Genomics.

[24] Andrews S. FastQC: a quality control tool for high throughput sequence data. 2010.

[25] Kim D, Langmead B, Salzberg SL (2015). HISAT: a fast spliced aligner with low memory requirements. Nat Methods.

[26] Anders S, Pyl PT, Huber W (2015). HTSeq–a Python framework to work with high-throughput sequencing data. Bioinformatics.

[27] Zerbino DR, Achuthan P, Akanni W, Amode MR, Barrell D, Bhai J (2018). Ensembl 2018. Nucleic Acids Res.

[28] Love MI, Huber W, Anders S (2014). Moderated estimation of fold change and dispersion for RNA-seq data with DESeq2. Genome Biol.

[29] Kono H, Rock KL (2008). How dying cells alert the immune system to danger. Nat Rev Immunol.

[30] Kim JB, Sig Choi J, Yu YM, Nam K, Piao CS, Kim SW (2006). HMGB1, a novel cytokine-like mediator linking acute neuronal death and delayed neuroinflammation in the postischemic brain. J Neurosci.

[31] Muhammad S, Barakat W, Stoyanov S, Murikinati S, Yang H, Tracey KJ (2008). The HMGB1 receptor RAGE mediates ischemic brain damage. J Neurosci.

[32] Kim T, Chelluboina B, Chokkalla AK, Vemuganti R (2019). Age and sex differences in the pathophysiology of acute CNS injury. Neurochem Int.

[33] Alkayed NJ, Murphy SJ, Traystman RJ, Hurn PD, Miller VM (2000). Neuroprotective effects of female gonadal steroids in reproductively senescent female rats. Stroke.

[34] Siegel C, Turtzo C, McCullough LD (2010). Sex differences in cerebral ischemia: possible molecular mechanisms. J Neurosci Res.

[35] Manwani B, Liu F, Scranton V, Hammond MD, Sansing LH, McCullough LD (2013). Differential effects of aging and sex on stroke induced inflammation across the lifespan. Exp Neurol.

[36] Lawrence T, Natoli G (2011). Transcriptional regulation of macrophage polarization: enabling diversity with identity. Nat Rev Immunol.

[37] Lee CK, Smith E, Gimeno R, Gertner R, Levy DE (2000). STAT1 affects lymphocyte survival and proliferation partially independent of its role downstream of IFN-gamma. J Immunol.

[38] Sironi JJ, Ouchi T (2004). STAT1-induced apoptosis is mediated by caspases 2, 3, and 7. J Biol Chem.

[39] Xu Q, Jiang C, Rong Y, Yang C, Liu Y, Xu K (2015). The effects of fludarabine on rat cerebral ischemia. J Mol Neurosci.

[40] Jung JE, Karatas H, Liu Y, Yalcin A, Montaner J, Lo EH (2015). STAT-dependent upregulation of 12/15-lipoxygenase contributes to neuronal injury after stroke. J Cereb Blood Flow Metab.

[41] Zhang MJ, Zhao QC, Xia MX, Chen J, Chen YT, Cao X (2020). The HDAC3 inhibitor RGFP966 ameliorated ischemic brain damage by downregulating the AIM2 inflammasome. FASEB J.

[42] He S, Liu R, Li B, Huang L, Fan W, Tembachako CR (2019). Propagermanium, a CCR2 inhibitor, attenuates cerebral ischemia/reperfusion injury through inhibiting inflammatory response induced by microglia. Neurochem Int.

[43] Yang Z, Ming XF (2014). Functions of arginase isoforms in macrophage inflammatory responses: impact on cardiovascular diseases and metabolic disorders. Front Immunol.

[44] Cai W, Dai X, Chen J, Zhao J, Xu M, Zhang L (2019). STAT6/Arg1 promotes microglia/macrophage efferocytosis and inflammation resolution in stroke mice. JCI Insight.

[45] Gray MJ, Poljakovic M, Kepka-Lenhart D, Morris SM (2005). Induction of arginase I transcription by IL-4 requires a composite DNA response element for STAT6 and C/EBPbeta. Gene.

[46] Sheldon KE, Shandilya H, Kepka-Lenhart D, Poljakovic M, Ghosh A, Morris SM (2013). Shaping the murine macrophage phenotype: IL-4 and cyclic AMP synergistically activate the arginase I promoter. J Immunol.

[47] Ocanas SR, Pham KD, Blankenship HE, Machalinski AH, Chucair-Elliott AJ, Freeman WM (2022). Minimizing the ex vivo confounds of cell-isolation techniques on transcriptomic and translatomic profiles of purified microglia. ENeuro..

[48] Masuda T, Amann L, Sankowski R, Staszewski O, Lenz M (2020). Novel Hexb-based tools for studying microglia in the CNS. Nat Immunol.

[49] Prinz M, Erny D, Hagemeyer N (2017). Ontogeny and homeostasis of CNS myeloid cells. Nat Immunol.

[50] Zhao Y, Mu H, Huang Y, Li S, Wang Y, Stetler RA (2022). Microglia-specific deletion of histone deacetylase 3 promotes inflammation resolution, white matter integrity, and functional recovery in a mouse model of traumatic brain injury. J Neuroinflammation.

[51] Auffray C, Sieweke MH, Geissmann F (2009). Blood monocytes: development, heterogeneity, and relationship with dendritic cells. Annu Rev Immunol.

[52] Chen HR, Sun YY, Chen CW, Kuo YM, Kuan IS, Tiger Li ZR (2020). Fate mapping via CCR2-CreER mice reveals monocyte-to-microglia transition in development and neonatal stroke. Sci Adv.

[53] Shi L, Rocha M, Zhang W, Jiang M, Li S, Ye Q (2020). Genome-wide transcriptomic analysis of microglia reveals impaired responses in aged mice after cerebral ischemia. J Cereb Blood Flow Metab.

[54] Xu M, Wang MM, Gao Y, Keep RF, Shi Y (2019). The effect of age-related risk factors and comorbidities on white matter injury and repair after ischemic stroke. Neurobiol Dis.

[55] Johnson S, Smith AG, Loffler H, Osby E, Juliusson G, Emmerich B (1996). Multicentre prospective randomised trial of fludarabine versus cyclophosphamide, doxorubicin, and prednisone (CAP) for treatment of advanced-stage chronic lymphocytic leukaemia. The French Cooperative Group on CLL. Lancet.

[56] Frank DA, Mahajan S, Ritz J (1999). Fludarabine-induced immunosuppression is associated with inhibition of STAT1 signaling. Nat Med.

[57] Plosker GL, Figgitt DP (2003). Oral fludarabine. Drugs.

[58] Foran JM, Oscier D, Orchard J, Johnson SA, Tighe M, Cullen MH (1999). Pharmacokinetic study of single doses of oral fludarabine phosphate in patients with "low-grade" non-Hodgkin's lymphoma and B-cell chronic lymphocytic leukemia. J Clin Oncol.

