# Supplementary material for: Targeted ablation of signal transducer and activator of transduction 1 alleviates inflammation by microglia/macrophages and promotes long-term recovery after ischemic stroke
Source: J Neuroinflammation. 2023 Jul 29;20:178. doi: 10.1186/s12974-023-02860-4 (PMC10385956; doi:10.1186/s12974-023-02860-4)
Supplement: Supplementary file 1 — Additional file 1: Table S1. Key resources. [file 12974_2023_2860_MOESM1_ESM.pdf]

Table S1. Key resources

REAGENT or RESOURCE	SOURCE	IDENTIFIER
**Antibodies**		
BUV395 Rat Anti-Mouse Ly-6G, Clone 1A8	BD Biosciences	563978
BUV737 Rat Anti-CD11b Clone M1/70	BD Biosciences	612800
CD45 Monoclonal Antibody (30-F11), PerCP-Cyanine5.5	eBioscience	45-0451-82
O4 Alexa Fluor 488-conjugated Antibody	R&D Systems	FAB1326G
APC-conjugated GLAST (ACSA-1) Antibody	Miltenyi Biotec	130-123-555
PE Mouse anti-Total Stat1 (N-Terminus)	BD Biosciences	558537
PE-conjugated GLAST (ACSA-1) Antibody	Miltenyi Biotec	130-118-344
Alexa Fluor 647 Mouse Anti-Stat1 (pY701)	BD Biosciences	612597
Rabbit anti-Iba1	Wako	019-19741
Rabbit polyclonal anti-MAP2	Millipore	AB5622-I
Rabbit polyclonal anti-NeuN	Millipore	ABN78
Rabbit polyclonal anti-HMGB1	Invitrogen	PA1-16926
Mouse Anti-NeuN Antibody, clone A60	Millipore	MAB377
Goat polyclonal anti-Iba1 antibody	abcam	ab5076
CD45 Monoclonal Antibody (30-F11), eFlour 450	eBioscience	48-0451-82
CD11b Monoclonal Antibody (M1/70), APC-eFlour 780	eBioscience	47-0112-82
BUV395 Rat Anti-Mouse Ly-6G, Clone 1A8	BD Biosciences	563978
CD11c Monoclonal Antibody (N418), PerCP-Cyanine5.5	eBioscience	45-0114-82
BUV737 Rat Anti-Mouse CD16/CD32 Clone 2.4G2	BD Biosciences	612783
TNF alpha Monoclonal Antibody (MP6-XT22), APC	eBioscience	17-7321-82
PE/Cyanine5 anti-mouse CD86 Antibody	BioLegend	105016
Brilliant Violet 605 anti-mouse CD206 (MMR) Antibody	BioLegend	141721
Human/Mouse Arginase 1/ARG1 PE-conjugated Antibody	R&D Systems	IC5868P
BV421 Rat Anti-Mouse IL-10 Clone JES5-16E3	BD Biosciences	563276
Rat Anti-Mouse CD16/CD32 (Mouse BD Fc Block) Clone 2.4G2	BD Biosciences	553142
Anti-arginase I Antibody (N-20)	Santa Cruz Biotechnology	sc-18351
BUV737 Rat Anti-Mouse CD19, Clone 1D3	BD Biosciences	612781
CD3 Monoclonal Antibody (17A2), APC	eBioscience	17-0032-82

F4/80 Monoclonal Antibody (BM8), PE	eBioscience	12-4801-82
Anti-Caspr Antibody, clone K65/35	Millipore	MABN69
Rabbit anti-NaV1.6 (SCN8A)	Alomone Labs	ASC-009
**Chemicals, Peptides, and Recombinant Proteins**		
Tamoxifen	Sigma-Aldrich	T5648
**Critical Commercial Assays**		
Mouse Inflammation Array C1	RayBiotech	AAM-INF-1
M.O.M. (Mouse on Mouse) Immunodetection Kit, Basic	Vector Laboratories	BMK-2202
In Situ Cell Death Detection Kit, TMR red	Roche	12156792910
Neural Tissue Dissociation Kit (T)	Miltenyi Biotec	130-093-231
OneComp eBeads Compensation Beads	ThermoFisher	01-1111-42
Zombie NIR Fixable Viability Kit	BioLegend	423105
eBioscience Foxp3/Transcription Factor Staining Buffer Set	Thermo Fisher	00-5523-00
Cell Lysis Buffer	Cell Signaling Technology	9803
Halt Protease and Phosphatase Inhibitor Cocktail	ThermoFisher	78842
RNeasy Plus Micro Kit	Qiagen	74034
RT2 First Strand Kit	Qiagen	330401
RT2 SYBR Green qPCR Mastermix	Qiagen	330502
SMART-Seq HT Kit	Takara Bio	634456
Nextera XT DNA Library Prep Kit Reference Guide	Illumina	15031942
**Experimental Models: Organisms/Strains**		
Mouse: C57BL/6J	The Jackson Laboratory	Stock No: 000664
Mouse: B6;129S-$Stat1^{tm1Mam}$/Mmjax	The Jackson Laboratory	MMRRC Stock No: 32054-JAX
Mouse: B6.129P2(Cg)-$Cx3cr1^{tm2.1(cre/ERT2)Litt}$/WganJ	The Jackson Laboratory	Stock No: 021160
**Oligonucleotides**		
Genotyping primer: $Cx3cr1^{CreER}$ common GAA CTA CAA TCC TTT AAG GCT CAC G	Ref. [1]	N/A
Genotyping primer: $Cx3cr1^{CreER}$ wild type GCA GGA CCT CGG GGT AGT CAC	Ref. [1]	N/A
Genotyping primer: $Cx3cr1^{CreER}$ mutant CAC CAG AGA CGG AAA TCC ATC G	Ref. [1]	N/A
Genotyping primer: $Stat1^{tm1Mam}$ GCA TGA CAT GAT CAG CAT TGC	The Jackson Laboratory	N/A
Genotyping primer: $Stat1^{tm1Mam}$ ACT GAC GTC AAC CAA GCC TG	The Jackson Laboratory	N/A
qPCR primer: Stat1 forward TCA CAG TGG TTC GAG CTT CAG	Ref. [2]	N/A
qPCR primer: Stat1 reverse GCA AAC GAG ACA TCA TAG GCA	Ref. [2]	N/A

qPCR primer: Gapdh forward GTG AAG GTC GGT GTG AAC GG	Ref. [3]	N/A
qPCR primer: Gapdh reverse GTT TCC CGT TGA TGA CCA G	Ref. [3]	N/A
**Deposited Data**		
Raw data files for RNA-seq	NCBI Gene Expression Omnibus	GSE141972<br>GSE234865
**Software and Algorithms**		
FlowJo	FlowJo, LLC	N/A
ImageJ	NIH	N/A
Imaris 9.6	Bitplane	N/A
Chipster v3.15	ref. [4]	https://chipster.csc.fi/
FastQC	ref. [5]	http://www.bioinformatics.babraham.ac.uk/projects/fastqc/
HISAT2	ref. [6]	https://github.com/DaehwanKimLab/hisat2
HTSeq	ref. [7]	http://htseq.readthedocs.io/
R v3.5	ref. [8]	https://www.r-project.org/
DESeq2	ref. [9]	http://bioconductor.org/packages/DESeq2/
Ingenuity Pathway Analysis	QIAGEN	N/A
Prism 9	GraphPad	N/A

References

1. Wang R, Pu H, Ye Q, Jiang M, Chen J, Zhao J, et al. Transforming Growth Factor Beta-Activated Kinase 1-Dependent Microglial and Macrophage Responses Aggravate Long-Term Outcomes After Ischemic Stroke. Stroke. 2020;51(3):975-85.

2. Simova J, Pollakova V, Indrova M, Mikyskova R, Bieblova J, Stepanek I, et al. Immunotherapy augments the effect of 5-azacytidine on HPV16-associated tumours with different MHC class I-expression status. Br J Cancer. 2011;105(10):1533-41.

3. Ishikawa K, Yoshida S, Nakao S, Sassa Y, Asato R, Kohno R, et al. Bone marrow-derived monocyte lineage cells recruited by MIP-1beta promote physiological revascularization in mouse model of oxygen-induced retinopathy. Lab Invest. 2012;92(1):91-101.

4. Kallio MA, Tuimala JT, Hupponen T, Klemela P, Gentile M, Scheinin I, et al. Chipster: user-friendly analysis software for microarray and other high-throughput data. BMC Genomics. 2011;12:507.

5. Andrews S. FastQC: a quality control tool for high throughput sequence data. 2010.

6. Kim D, Langmead B, Salzberg SL. HISAT: a fast spliced aligner with low memory requirements. Nat Methods. 2015;12(4):357-60.

7. Anders S, Pyl PT, Huber W. HTSeq--a Python framework to work with high-throughput sequencing data. Bioinformatics. 2015;31(2):166-9.

8. R Core Team. R: A language and environment for statistical computing. Vienna, Austria: R Foundation for Statistical Computing; 2020.

9. Love MI, Huber W, Anders S. Moderated estimation of fold change and dispersion for RNA-seq data with DESeq2. Genome Biol. 2014;15(12):550.
